# Supplementary material for: Resistance to insect growth regulators and age-stage, two-sex life table in Musca domestica from different dairy facilities
Source: PLoS One. 2021 Apr 8;16(4):e0248693. doi: 10.1371/journal.pone.0248693 (PMC8031857; doi:10.1371/journal.pone.0248693)
Supplement: Attachment — Submitted filename: PONE-D-20-31395_R1_reviewer.pdf [file pone.0248693.s007.pdf]

PLOS ONE

Resistance to insect growth regulators and age-stage, two-sex life table in Musca domestica from different dairy facilities

--Manuscript Draft--

**Manuscript Number:**	PONE-D-20-31395R1
**Article Type:**	Research Article
**Full Title:**	Resistance to insect growth regulators and age-stage, two-sex life table in Musca domestica from different dairy facilities
**Short Title:**	Resistance to insect growth regulators and life table of house fly
**Corresponding Author:**	Naeem Abbas<br>King Saud University College of Food and Agricultural Sciences<br>Riyadh, SAUDI ARABIA
**Keywords:**	Livestock pest; biological traits; ecology; toxicity; age-specific survivorship; integrated vector management
**Abstract:**	Among the vectorial insect pests, the domestic house fly (Musca domestica L., Diptera: Muscidae) is a ubiquitous livestock pest with the ability to develop resistance and adapt to diverse climates. Successful management of the house fly in various locations requires information about its resistance development and life table features. The status of insect growth regulators resistance and life table features on the basis of age, stage, and two sexes of the house fly from five different geographical locations of Riyadh, Saudi Arabia: Dirab, Al-Masanie, Al-Washlah, Al-Uraija and Al-Muzahmiya were therefore investigated. The range of resistance levels were 3.77–8.03-fold for methoxyfenozide, 5.50–29.75 for pyriproxyfen, 0.59–2.91-fold for cyromazine, 9.33–28.67-fold for diflubenzuron, and 1.63–8.25-fold for triflumuron in five populations of house fly compared with the susceptible strain. Analysis of life history parameters—such as survival rate, larval duration, pupal duration, pre-female duration, pre-male duration, adult and total pre-oviposition periods, longevity of male, oviposition days, female ratio, and fecundity female -1 —revealed significant variations among the field populations. Additionally, demographic features—including the generation time, the finite and intrinsic rates of increase, doubling time, and net reproductive rate—varied significantly among the field populations. These results will be helpful in planning the management of the house fly in geographically isolated dairies in Saudi Arabia.
**Order of Authors:**	Naeem Abbas
	Abdulwahab M Hafez
**Response to Reviewers:**	PONE-D-20-31395<br>Resistance to insect growth regulator insecticides and age-stage, two-sex life table parameters in house fly, Musca domestica from different dairy facilities<br><br>1. Please ensure that your manuscript meets PLOS ONE's style requirements, including those for file naming. The PLOS ONE style templates can be found at https://journals.plos.org/plosone/s/file?id=wjVg/PLOSOne_formatting_sample_main_body.pdf and https://journals.plos.org/plosone/s/file?id=ba62/PLOSOne_formatting_sample_title_authors_affiliations.pdf<br>Answer: Carefully checked for style<br>2. In your Methods section, please provide additional information regarding the permits you obtained for the work. Please ensure you have included the full name of the authority that approved the collection sites access and, if no permits were required, a brief statement explaining why.<br>Answer: Ethic statement added in the manuscript<br><br>Additional Editor Comments:<br>The article is interesting and seems useful in managment of housefly on a worldwide scale. The methods and data seem basically sound. However, the article still needs some modifications to increase readability. There is a mismatch between text and

Powered by Editorial Manager® and ProduXion Manager® from Aries Systems Corporation

tables where the sample size in the tables exceeds that expected based on the text. In addition, language improving is needed.
Answer: Corrected and English language carefully checked and improved. The manuscript is edited for English language and spelling by Enago, an editing brand of Crimson Interactive Inc.
Reviewer #1: This is a review of the paper entitled "Resistance to insect growth regulator insecticides and age-stage, two-sex life table parameters in house fly, Musca domestica from different dairy facilities." The research looked at resistance development for five insecticides in the house fly at five dairy farms in Saudi Arabia. The research is useful in documenting the current state of resistance.
1) English could be improved. It was clear enough that I felt I understood the manuscript, but there were significant problems.
Answer: Thanks for comment, Carefully checked and improved
2) Expand thoughts into sentences. Line 50 is an example.
Original: "The study of life tables on the theory of age, stage, and two sexes of insect pests …"
Problems: Studying life tables is the start of a scientific discipline called demography. Studying the tables without underlying theory is of little use. There is no theory of "age, stage, and two sexes."
One solution: Demography is a basic and important tool in pest management [22-24].
Another solution) The study of life tables involves summarizing age or stage specific birth, death, and reproductive data. Classic models typically focus on females, but we will use both males and females. This methodology is a basic tool in pest management [22-24].
Answer: Done as suggested
3) It would help to put the insecticides into their IRAC classification: pyriproxyfen (7C), triflumuron and diflubenzuron (15), cyromazine (17), and methoxyfenozide (18).
Answer: Done as suggested
4) Maybe put the insecticides in order. The order could be alphabetical, or in order of increasing/decreasing activity, or by IRAC classification. Alphabetical is my least favorite. Assertions later in the manuscript suggest ordering by elevation or latitude/longitude.
Answer: Done by IRAC classification
5) The authors calculate all the variables that they can find, but it is difficult to grasp an overall sense of what these differences mean. Would the authors consider a final figure that shows the estimated population growth over several generations? That way we can see how early reproduction versus many offspring plays out over time. If it makes sense the model could be driven by a degree-day approach to further customize outcomes to environmental conditions at each dairy.
Answer: Thanks for comment, unfortunately we did not take the data of population growth for several generations, already a big set of data is presented in the manuscript but we will consider in future experiments.
6) Tables and text MUST agree. The text (Lines 99-110) state that an assay consisted of 5-concentrations, 4-replicates, 10-larvae = 200. The control had 4-replicates of 10-larvae = 40. N in table 1 has 240, or 280 for treatments, and 210, or 270 for the controls.
Answer: Corrected and cleared
7) The data analysis is incomplete. Tables 2 and 3 have letters typically used in some type of multiple comparison procedure. The table footnote indicates a "paired bootstrap test" but no further details are given. Does the test correct for the experiment wise error rate, or is it a bootstrap version of a t-test that should have had a Bonferroni correction applied?
Answer: Data were analysed by paired bootstrap test using TWO-SEX MS chart program. Lettering is done manually based on confidence intervals difference and P value (<0.05). Standard errors of means (SEM) were estimated using bootstrapping (100,000 re-samplings) using TWOSEX MS chart program.
8) Is there any record of current practices at these dairies? What they have used in the past, application frequency, that sort of thing?
Answer: Added in material and method section
Specific comments:
Line 34) "capable to adapt" change to "capable of adapting to"
Answer: Done as suggested
Line 34) "This insect is"
Answer: Done as suggested

Line 34) “as the house”
Answer: Done as suggested
Line 35) change serve to serves
Answer: Done as suggested
Line 36) Attempts to control the house fly with a wide range of insecticides has failed because this insect rapidly develops resistance to these chemicals.
Answer: Done as suggested
Line 41-42) “comparatively environmentally safe” is a sentence fragment.
Answer: Rephrased
Line 50) “The study of life tables on the theory of age, …”
Answer: Rephrased
Line 64) depends
Answer: Done as suggested
Line 64) If true then livestock/urban pest control professionals need to read more of the resistance management literature from agriculture where mode of action rotations, and other methods have been developed. “Environmental factors” as resistance management tools probably would not even make the list of most tree and row crops producers. However, the difference between open versus closed systems is a good point. Closed systems are becoming more popular in the agricultural sector whether entirely artificial or screened enclosures.
Answer: Rephrased
Line 78) I would remove “after attraction by a mixture of dried milk and sugar” and make this text a new sentence. As written, I might think that the milk and sugar came from five dairy farms.
Answer: Removed as suggested
Line 84, 101, 123, and elsewhere) (37] Why a mix of round and square brackets?
Answer: Corrected
Line 86) This is an unanswerable question with these data, but how quickly is resistance lost under zero-stress laboratory conditions? If there is a competitive disadvantage to resistance in the absence of insecticide then the reported values are some lower bound to the true value.
Answer: Human error, the populations were reared for one generation, corrected
Line 88) delete “after”
Answer: Done
Line 89) delete “with a micro-syringe (5 ml)” In this case the issue is that the use of a micro-syringe is unimportant detail and it is unclear if the syringe volume was 5 ml or you used the syringe to deliver 5 ml to the wick. In either case the volume of water needed will depend on the number of flies, temperature, and humidity in the cage.
Answer: Deleted
Paragraph line 99) How was this put together? I could run 1 cup at each concentration every week, or I could make a large batch of diet and fill all 4 cups of each concentration at once. Were the larvae of same age or different ages? Were the larvae still alive? What fraction were alive versus dead?
Answer: Bioassay for each insecticide and each population were conducted separately. Fresh solution was made for every population. Same age (2nd instar) larvae were used. Diet was mixed with a concentration for 4 replications together and then equally provided in four cups. Alive larvae were fed on the treated diet till pupation. Data was recorded on adult emergence. We did not noted fraction in larval stage, we noted emerged adults, from this mortality was calculated, For example, for one concentration 40 larvae were exposed, out of this 4 adults emerged, it means 36 larvae were died for that concentration and so on…
Line 112) “100 freshly laid eggs at different days were randomly…” This sentence is confusing. I will guess that you found freshly laid (define fresh) egg masses and arbitrarily removed one egg per egg mass. This was done over several days until you had 100 individuals.
Answer: Rephrased
Line 115) 3 does not go into 100 evenly.
Answer: Rephrased
Line 117) Freshly emerged adults within 24 h were sexed and one male and one female placed into plastic jars (15 cm x 11 cm).
Answer: Done
121) You do not conduct parameters under laboratory conditions.
Answer: Corrected
Line 140) The equations are meaningless without indicating what all the symbols

Powered by Editorial Manager® and ProduXion Manager® from Aries Systems Corporation

mean. Some definitions are in the tables.
Answer: Added
Line 127) follows not follow
Answer: Done
Line 127) Ro is also sometimes Ro, R0 or are these all different?
Equations) There are odd mistakes in the equations. If A=b and b=1 then does not A=1? The equation for T is simply wrong. Use parentheses as appropriate to make things clearer. For example, sqrtx should not be written when you mean sqrt(x). “sqrtx” is a variable name, while “sqrt(x)” indicates applying sqrt to a variable “x”. As written I need a definition for “In” and I need a definition for “Ro” and I am missing an operator telling me what to do with these (add, subtract, multiple, divide, something else..).
Answer: Ro similar, Corrected
Line 149) The web link for citation 42 did not work. It returns “can’t reach this page” error.
Answer: New version available at: http://140.120.197.173/Ecology/prod02.htm
Line 147) do not use tolerance. Tolerance has a specific definition in host plant resistance. In this context it is confusing. If the insect tolerates the insecticide is it not resistant? Low-level resistance is clear enough as it is unclear if low-level resistance is significantly different from susceptible. In some cases yes (methoxyfenozide) and other cases sometimes. For pyriproxyfen no for Al0Washlah, but yes for Al-Muzahmiya based on overlap of the 95% FL.
Answer: Deleted
Line 156) Do not use tolerance.
Answer: Deleted
Line 167) “different from” not “different to”
Answer: Done
Line 169) What is pre-male? Do you mean male larvae? I assume that you sex the adults and work backwards.
Answer: egg to adult duration for male, changed
Line 169-170) “The pre-male duration of the Al-Masanie” is more clearly written as “The egg to adult duration for male flies from the Al-Masanie population was significantly longer than for flies from all other areas except Dirab.”
Answer: Done as suggested
Line 171) pre-female same problem as with pre-male.
Answer: Changed as suggested for male
Line 177) Avoid acronyms whenever possible. A) In a table where space is limited use acronyms and define in footnotes. B) TPOP for total pre-oviposition period is a good acronym if you need to use it dozens of times. If the goal is to make it easier for readers to find relevant parts in tables/figures then define at each use in text: total pre-oviposition period (TPOP) …
Answer: Change as suggested
Table 1) The locations are in different order for each insecticide. Why?
Answer: Changed and made consistent for each insecticide
Table 1) Why are the chi-square degrees of freedom sometime 3, 4, or 6?
Answer: Corrected
Table 1) Why is N sometimes 210, 240, 270, or 280?
Answer: Human error, corrected
Table 2) For Al-Washlah: N=8 for TPOP yet N=20 for oviposition. I have magically gained 12 individuals. There are similar problems elsewhere.
Answer: Corrected, total females were 20, and 9 females produced eggs
Figure 1) No clue why some area is clear other colored.
Answer: Corrected
Figure 1) The figures look more like an age/stage decomposition of survival where “death” in one stage can be mortality or “birth” into the next stage. However, “birth” rate in this sense is not exactly survival rate. That said, the figures are already hard to read and another set of points will make that problem worse. The individual graphs have considerable white space. Could a small inset of “total survival” be added?
Answer: Added
Figure 3) Filled areas obscure parts of the graph.
Answer: Corrected
Line 212) “house flies from dairies” is a better choice than dairy house flies because it is clearer that these are house flies that happen to be collected at dairies.
Answer: Changed
Line 262) “found” rather than “occurred”

Powered by Editorial Manager® and ProduXion Manager® from Aries Systems Corporation

Answer: Done
Line 269) That is a major point in the insecticide resistance action committee (IRAC) work. https://irac-online.org/
Answer: Added
Line 312) Does the range in latitude/longitude in this study warrant such attribution relative to the range in the cited studies? If the study sites are arranged by latitude/longitude would they support this hypothesis? This is a large enough piece of work that you do not need to speculate on unfounded hypotheses. Besides, if the real answer is latitude/longitude then there is nothing that these farmers can do to solve their problem.
Answer: Deleted
Line 312) How would you separate the effect of latitude/longitude from geographic distance? The dairies that are 2-kilometers distant will have more in common than ones that are 2000-km distant.
Line 312) if the dairies are ordered by elevation does a pattern emerge? You have five sites and five estimates of r, does a linear regression show a relationship between r and elevation in your data? If not, then this is pointless speculation that can be deleted.
Answer: Deleted

Review report:
Manuscript Number: PONE-D-20-31395
Full Title: Resistance to insect growth regulator insecticides and age-stage, two-sex life table
parameters in house fly, Musca domestica from different dairy facilities

Comments:
The topic of the article is interesting and bears implications in management of housefly (Musca domestica) on a worldwide scale. Although the present study considered the housefly from dairy facilities, still, the information is useful for the population regulation of housefly elsewhere where the insecticide resistance is a problem. The study design including the experiments and statistical analyses and the compilation appears to be suitable for addressing the issues of insecticide resistance of housefly. However, few minor corrections are required with reference to the language and the title of the article.
Suggestions:
1] Please consider a concise title of this article.
Answer: Done
2]The hypothesis of your study should be mentioned clearly at the end of the introduction section.
Answer: Done
3]Please use an alternative to life table traits…you can consider life table features.
Answer: Done
4]Please check for the typographical errors if any.
Answer: Checked and removed
5] Please consider the following articles for inclusion in discussion section:
Kočišová A, Petrovský M, Toporčák J, Novák P. 2004. The potential of some insect growth regulators in housefly (Musca domestica) control. Biologia, 59/5: 661—668. (Please consider this article as important to compare and highlight the impact of the insect growth regulators in housefly regulation. Please consider inclusion of the content of this paper in the introduction section.)
Malik A, Singh N, Satya S. 2007. House fly (Musca domestica): A review of control strategies for a challenging pest. Journal of Environmental Science and Health, Part B, 42:4, 453-469. [Please consider this article as important to highlight the various control measures available….both in the introduction and discussion sections]
Farooq M, Freed S. 2016. Infectivity of housefly, Musca domestica (Diptera: Muscidae) to different entomopathogenic fungi. Brazilian Journal of Microbiology, 47: 807 -816.
[Please consider this article for highlighting the fungal agents in biocontrol of housefly]

Abbas N, & Khan A, Shad, S. 2014. Resistance of the house fly Musca domestica (Diptera: Muscidae) to lambda-cyhalothrin: Mode of inheritance, realized heritability, and cross-resistance to other insecticides. Ecotoxicology (London, England). 23. 10.1007/s10646-014-1217-7. [Please consider this article for the elaboration of resistance to pesticides by the housefly]

Answer: Added above references as suggested

Powered by Editorial Manager® and ProduXion Manager® from Aries Systems Corporation

	6] Please improve the quality of the figures, particularly, Figure 3.<br>Answer: Improved
**Additional Information:**	
**Question**	**Response**
**Financial Disclosure**<br><br>Enter a financial disclosure statement that describes the sources of funding for the work included in this submission. Review the submission guidelines for detailed requirements. View published research articles from *PLOS ONE* for specific examples.<br><br>This statement is required for submission and **will appear in the published article** if the submission is accepted. Please make sure it is accurate.<br><br>**Unfunded studies**<br>Enter: *The author(s) received no specific funding for this work.*<br><br>**Funded studies**<br>Enter a statement with the following details:<br>• Initials of the authors who received each award<br>• Grant numbers awarded to each author<br>• The full name of each funder<br>• URL of each funder website<br>• Did the sponsors or funders play any role in the study design, data collection and analysis, decision to publish, or preparation of the manuscript?<br>• **NO** - Include this sentence at the end of your statement: *The funders had no role in study design, data collection and analysis, decision to publish, or preparation of the manuscript.*<br>• **YES** - Specify the role(s) played.<br><br>* typeset	The authors extend the appreciation for the Deanship of Scientific Research at King Saud University for funding this work through the research group No. RGP-1441-480. The funders had no role in study design, data collection and analysis, decision to publish, or preparation of the manuscript.
**Competing Interests**<br><br>Use the instructions below to enter a competing interest statement for this submission. On behalf of all authors, disclose any competing interests that could be perceived to bias this work—acknowledging all financial support and any other relevant financial or non-	The authors have declared that no competing interests exist

financial competing interests.<br><br>This statement **will appear in the published article** if the submission is accepted. Please make sure it is accurate. View published research articles from *PLOS ONE* for specific examples.<br><br>**NO authors have competing interests**<br><br>Enter: *The authors have declared that no competing interests exist.*<br><br>**Authors with competing interests**<br><br>Enter competing interest details beginning with this statement:<br><br>*I have read the journal's policy and the authors of this manuscript have the following competing interests: [insert competing interests here]*<br><br>* typeset	
**Ethics Statement**<br><br>Enter an ethics statement for this submission. This statement is required if the study involved:<br><br>• Human participants<br>• Human specimens or tissue<br>• Vertebrate animals or cephalopods<br>• Vertebrate embryos or tissues<br>• Field research<br><br>Write "N/A" if the submission does not require an ethics statement.<br><br>General guidance is provided below. Consult the submission guidelines for detailed instructions. **Make sure that all information entered here is included in the Methods section of the manuscript.**	N/A

Powered by Editorial Manager® and ProduXion Manager® from Aries Systems Corporation

**Format for specific study types**<br><br>**Human Subject Research (involving human participants and/or tissue)**<br>• Give the name of the institutional review board or ethics committee that approved the study<br>• Include the approval number and/or a statement indicating approval of this research<br>• Indicate the form of consent obtained (written/oral) or the reason that consent was not obtained (e.g. the data were analyzed anonymously)<br><br>**Animal Research (involving vertebrate animals, embryos or tissues)**<br>• Provide the name of the Institutional Animal Care and Use Committee (IACUC) or other relevant ethics board that reviewed the study protocol, and indicate whether they approved this research or granted a formal waiver of ethical approval<br>• Include an approval number if one was obtained<br>• If the study involved *non-human primates*, add *additional details* about animal welfare and steps taken to ameliorate suffering<br>• If anesthesia, euthanasia, or any kind of animal sacrifice is part of the study, include briefly which substances and/or methods were applied<br><br>**Field Research**<br><br>Include the following details if this study involves the collection of plant, animal, or other materials from a natural setting:<br>• Field permit number<br>• Name of the institution or relevant body that granted permission	
**Data Availability**<br><br>Authors are required to make all data underlying the findings described fully available, without restriction, and from the time of publication. PLOS allows rare exceptions to address legal and ethical concerns. See the PLOS Data Policy and FAQ for detailed information.	Yes - all data are fully available without restriction

A Data Availability Statement describing where the data can be found is required at submission. Your answers to this question constitute the Data Availability Statement and **will be published in the article**, if accepted.<br><br>**Important:** Stating 'data available on request from the author' is not sufficient. If your data are only available upon request, select 'No' for the first question and explain your exceptional situation in the text box.<br><br>Do the authors confirm that all data underlying the findings described in their manuscript are fully available without restriction?	
**Describe where the data may be found in full sentences. If you are copying our sample text, replace any instances of XXX with the appropriate details.**<br><br>• If the data are **held or will be held in a public repository**, include URLs, accession numbers or DOIs. If this information will only be available after acceptance, indicate this by ticking the box below. For example: *All XXX files are available from the XXX database (accession number(s) XXX, XXX.)*.<br>• If the data are all contained **within the manuscript and/or Supporting Information files**, enter the following: *All relevant data are within the manuscript and its Supporting Information files.*<br>• If neither of these applies but you are able to provide **details of access elsewhere**, with or without limitations, please do so. For example:<br><br>*Data cannot be shared publicly because of [XXX]. Data are available from the XXX Institutional Data Access / Ethics Committee (contact via XXX) for researchers who meet the criteria for access to confidential data.*<br><br>*The data underlying the results presented in the study are available from (include the name of the third party*	All relevant data are within the manuscript and its Supporting Information files.

and contact information or URL).<br>• This text is appropriate if the data are owned by a third party and authors do not have permission to share the data.<br><br>* typeset	
Additional data availability information:	

Manuscript

Click here to access/download;Manuscript;Manuscript_18-12-20.docx

Resistance to insect growth regulators and age-stage, two-sex life table in *Musca domestica* from different dairy facilities

Naeem Abbas* and Abdulwahab M. Hafez*

Pesticides and Environmental Toxicology Laboratory, Department of Plant Protection, College of Food and Agriculture Sciences, King Saud University, P.O. Box 2460, Riyadh 11451, Saudi Arabia

*Corresponding authors:

Email: nnoor.c@ksu.edu.sa, ORCID: 0000-0002-2737-7487 (NA)

Email: hafez@ksu.edu.sa, ORCID: 0000-0003-4290-5548 (AH)

Abstract

Among the vectorial insect pests, the domestic house fly (*Musca domestica* L., Diptera: Muscidae) is a ubiquitous livestock pest with the ability to develop resistance and adapt to diverse climates. Successful management of the house fly in various locations requires information about its resistance development and life table features. The status of insect growth regulators resistance and life table features on the basis of age, stage, and two sexes of the house fly from five different geographical locations of Riyadh, Saudi Arabia: Dirab, Al-Masanie, Al-Washlah, Al-Uraija and Al-Muzahmiya were therefore investigated. The range of resistance levels were 3.77–8.03-fold for methoxyfenozide, 5.50–29.75 for pyriproxyfen, 0.59–2.91-fold for cyromazine, 9.33–28.67-fold for diflubenzuron, and 1.63–8.25-fold for triflumuron in five populations of house fly compared with the susceptible strain. Analysis of life history parameters—such as survival rate, larval duration, pupal duration, pre-female duration, pre-male duration, adult and total pre-oviposition periods, longevity of male, oviposition days, female ratio, and fecundity female^{-1}—revealed significant variations among the field populations. Additionally, demographic features—including the generation time, the finite and intrinsic rates of increase, doubling time, and net reproductive rate—varied

significantly among the field populations. These results will be helpful in planning the management of the house fly in geographically isolated dairies in Saudi Arabia.

Key words: Livestock pest; biological features; ecology; toxicity; age-specific survivorship; integrated vector management

Introduction

Musca domestica L. (Diptera: Muscidae) is a ubiquitous insect pest of livestock and humans [1, 2] capable of adapting to a wide range of climates. This insect is commonly known as the house fly and serves as a carrier for many pathogens of public health and veterinary importance [3, 4]. Attempts to control the house fly with a wide range of insecticides have failed because this insect rapidly develops resistance to these chemicals [5-11]. This widespread development of insecticide resistance has necessitated the employment of integrated vector management strategies.

Various control measures are available to manage the house fly, for example, cultural practices, chemicals, and biological agents including fungal/bacterial pathogens and parasitoids/predators or combination of these measures [12, 13]. Insect growth regulators (IGR) comprise an effective chemical tool for integrated pest management due to their properties of high selectivity, killing immature stages, comparative environmentally safe, and reduced risk to the safety of mammals [14-16]. The juvenile hormone mimic pyriproxyfen inhibits the emergence of adults, cyromazine is a molting disrupter, diflubenzuron and triflumuron are inhibitors of chitin biosynthesis affecting CHS1, and methoxyfenozide is an ecdysone agonist [17]. These insecticides are effectively recommended to manage medical pests, including *M. domestica*, and are strong candidates for integrated pest management due to their different novel mode of actions and less environmental hazards [16, 18-20]. However, resistance to IGRs has been reported previously in house fly [20-25].

The study of life tables involves summarizing age or stage specific birth, death, and reproductive data. Classic models typically focus on females, but we will use both males and females. This methodology is a basic tool in pest management [26-28]. Comparison of the life tables of geographically separated pest populations aids in the design of effective pest management strategies. Geographic influences such as latitude and longitude and environmental influences such as humidity, photoperiod, and temperature often account for variations in the life tables of insect pests [29, 30]. Geographically and environmentally isolated pest populations have previously been reported to show variations in life tables, for instance, the house fly from urban localities in Pakistan [31]; *Parapoynx crisonalis* (Walker) (Lepidoptera: Pyralidae) [29]; *Plutella xylostella* (Linnaeus) (Lepidoptera: Plutellidae) from China [32]; and *Melanoplus femurrubrum* (De Geer) (Orthoptera: Acrididae) from USA [33]. Variations have also emerged in pesticide resistance in the house fly, *Anopheles stephensi* Liston (Diptera: Culicidae); *Oxycarenus hyalinipennis* Costa (Hemiptera: Lygaidae); *Amrasca devastans* (Distant) (Hemiptera: Cicadellidae); and *Dysdercus koenigii* (Fabricius) (Hemiptera: Pyrrhocoridae) [5, 7, 20, 34-39].

Development of resistance in spatial dispersed pests is important to consider in resistant management strategies. For example, the populations of house fly from Punjab, Pakistan showed significant variations in resistant levels to various insecticides under different environments such as dairy (open) and poultry farms (enclosed) [5, 40]. The knowledge of insecticide resistance and life table variations in the house fly from geographically separated dairies is very important in the design of effective management plans in different regions. Resistance to insect growth regulator insecticides and variations in the life history features of house fly from different originated dairies in Saudi Arabia are currently unexplored. Therefore, the present experiment was conducted to obtain an

understanding of the resistance status and life table on the basis of age, stage and two sexes of the house fly collected from different dairies in Saudi Arabia.

Materials and methods

Ethics approval

No specific permit was required to collect house fly samples from the dairy farms, as these farms were privately owned and collections were made merely by personal communication with the owners.

Collections of house fly populations

Approximately 150-200 adult house flies (of mixed sexes) were trapped in plastic jars (33 cm ×19 cm) from five dairy farms located in Riyadh, Saudi Arabia: Dirab (latitude 24.49° N, 46.62° E; elevation 576 m), Al-Masanie (latitude 24.58° N, 46.73° E; elevation 581 m), Al-Uraija (latitude 24.63° N, 46.66° E; elevation 612 m), Al-Washlah (latitude 24.54° N, 46.72° E; elevation 643 m), and Al-Muzahmiya (latitude 24.47° N, 46.27° E; elevation 764 m). At these dairies, cultural practices (sanitation) and about 8-10 number of insecticide sprays from pyrethroids, organophosphates, and new chemistry classes per season are being used for the control of dairy pests (Personal communication).

Laboratory rearing of house fly populations

The rearing protocol of the house fly was adopted according to Abbas et al. [41] with some modifications. After collection, each population was shifted in a separate transparent cage (40×40 cm) in the laboratory and reared for one generation to get uniform insects. An adult diet (sugar + powdered milk at a 1:1 ratio, weighed in g) and water-soaked cotton wicks placed in 9 cm plastic petri dishes were provided for adult food. Adults were provided with fresh food every two days. Cotton wicks were moistened daily and replaced at two days

interval. A paste of wheat bran, yeast, sugar and milk at a ratio of 20:5:1.5:1.5 (g), respectively, mixed with 120 mL water, was prepared in plastic cups (500 ml) and placed in adult cages for fecundity after two days rearing in the laboratory. Plastic cups with eggs were removed from adult cages daily and covered with a muslin cloth to avoid larval escape. When larvae had consumed the diet in the plastic cups, they were shifted into glass beakers with larval medium, where they became pupae. The emerged adults were shifted to cages to proceed to the next generation. All populations were maintained at 27±2 ºC, 65±5% humidity and a 12:12 h (L:D) photoperiod in the laboratory.

Insecticides

Commercial-grade formulated IGR groups including pyriproxyfen (IRAC # 7C, Admiral 10EC, Sumitomo Chemicals, Japan), diflubenzuron (IRAC # 15, Diflon 250WP, Saudi Delta Company, SA), triflumuron (IRAC # 15, Starycide 480SC, Bayer Crop Sciences, Germany), cyromazine (IRAC # 17, Novasat 75WP, Astranova Chemicals, SA), and methoxyfenozide (IRAC # 18, Runner 24SC, Dow Agro-sciences, UK).

Larval bioassays

The toxicity of IGR insecticides to the larvae of different dairy populations of *M. domestica* was determined through a diet incorporation bioassay following the method of Abbas et al. [20]. Different concentrations of each of the tested IGRs were incorporated into the larval medium (consisting of wheat bran, yeast, milk powder, and sugar at a ratio of 20:5:5:1.5:1.5 g, respectively). Five concentrations (causing mortality from >0% to <100%) of each IGR with four replicates of each were used in each bioassay. In total, 10 second instar larvae in each replicate, 40 larvae in each concentration, and 200 larvae were used in each bioassay. In the control treatment, 40 larvae with four replicates (10 larvae per replicate) were provided with

larval medium without any IGR insecticide concentrations. All bioassays were conducted under the above mentioned constant conditions. Data were recorded at the emergence of adults after 3 weeks of the bioassay. Larvae that failed to transform into adults were considered as dead.

Life table construction

To construct the life table of house fly, 100 newly laid eggs were randomly collected from different egg batches at one day of each mass population and were placed in plastic cups (500 ml) supplied with artificial larval diet aforementioned above. Single egg was considered as a replicate for each population [42]. The cups were enclosed with muslin cloth to prevent larval escape. Each population was reared separately. Hatched larvae were reared in cups on provided diet until pupation, and their developmental periods were recorded. Freshly emerged adults within 24 h were sexed and one male and one female placed into plastic jars (15 cm x 11 cm). Larval medium was provided daily in 9 cm petri dishes for oviposition. Eggs were counted daily till the death of females and egg hatchability was recorded. The female ratio, adult longevity, and oviposition days were also recorded. The experiment was conducted under the aforesaid laboratory conditions.

For each strain, the following parameters were calculated according to Chi and Su [43] and Tuan et al. [27]. Briefly, the age-specific ~~survival rate~~ (l_x) was determined as follows:

$$l_x = \sum_{j=1}^{m} s_{xj}$$

The age-stage-specific fecundity (m_x) was determined as follows:

$$m_x = \frac{\sum_{j=1}^{m} s_{xj} f_{xj}}{\sum_{j=1}^{m} s_{xj}}$$

The net reproductive rate (~~Ro~~) was calculated as follows:

$$R_O = \sum_{x=1}^{\infty} l_x m_x$$

The intrinsic rate of increase (r) was assessed by the Lotka–Euler equation with the age indexing zero as follows:

$$r = \sum_{x=0}^{\infty} e^{r(x+1)} l_x m_x = 1$$

The finite rate of increase (λ) was determined as:

$$\lambda = e^r$$

The generation time (T) was determined as follows:

$$T = \frac{\text{In (Ro)}}{r}$$

The life expectancy (e_{xj}) was calculated as follows:

$$e_{xj} = \sum_{i=x}^{n} \sum_{j=y}^{m} s'_{ij}$$

The reproductive value (v_{xj}) was detected as follows:

$$v_{xj} = \frac{e^{-r(x+1)}}{s_{xj}} \sum_{i=x}^{n} e^{-r(i+1)} \sum_{j=y}^{m} s'_{ij} f_{ij}$$

The gross reproduction rate (GRR) was calculated as:

$$GRR = \sum m_x$$

Data analyses

The bioassay data were analyzed using the POLO Plus software [44] to determine the values of median lethal concentration (LC_{50}). In each bioassay, the mortality rates were corrected by the mortality rate obtained in the control treatment using the formula of Abbott [45], if required. Resistance ratios (RRs) were calculated as: LC_{50} of field population/LC_{50} of susceptible strain. The resistance levels were scaled as mention by Ahmad et al. [46]; RR = 1

(susceptibility), RR = 2–10 (low resistance), RR = 11–30 (moderate resistance), RR = 31–100 (high resistance), and RR > 100 (very high resistance).

The life table data were analyzed with the TWO-SEX-MS Chart program [47] established on the basis of two sex life table principle [28, 48]. The variances and standard errors (SE) of life history features were determined by paired bootstrap test with 100,000 replicates at $P \leq 0.05$ using the TWOSEX-MS Chart [47]. The parameters l_x, s_{xj}, f_x, m_x, $l_x m_x$, v_{xj}, and e_{xj} were graphed with Sigma Plot 11.0.

Results

Resistance of *M. domestica* larvae to IGRs

Low resistance against methoxyfenozide (3.77–8.03-fold) and triflumuron (1.63–8.25-fold) was detected in all the five populations of *M. domestica* in comparison to the susceptible strain. Populations collected from the Dirab, Al-Masanie, and Al-Uraija had moderate resistance (10.75–29.75-fold) to pyriproxyfen, while the Al-Washlah and Al-Muzahmiya populations had low resistance levels (5.50–8.25-fold), compared with the susceptible strain. Susceptibility to cyromazine was found in all the tested populations (0.59–1.32-fold), except the Al-Masanie population which had low resistance (2.91-fold). Populations collected from the Dirab, Al-Washlah, Al-Masanie, and Al-Muzahmiya had moderate resistance (13.67–28.67-fold) to diflubenzuron, while the Al-Uraija population had low resistance level (9.33-fold), compared with the susceptible strain (Table 1).

Life history parameters of house flies from dairies

The larval durations of all the tested populations were significantly different from each other ($P < 0.05$). The pupal duration of the Al-Uraija population was significantly longer than that of the Dirab, Al-Masanie, Al-Washlah and Al-Muzahmiya populations ($P < 0.05$). The egg to adult duration for male flies from the Al-Masanie population was significantly longer than for flies from all other areas except Dirab ($P < 0.05$). Similarly, the egg to adult duration for

female flies from the Al-Masanie population was significantly longer than for flies from all other areas ($P < 0.05$). The male total longevity in the Al-Uraija and Al-Muzahmiya populations was significantly shorter than those of the Dirab, Al-Masanie, and Al-Washlah populations ($P < 0.05$). The female total longevity in the Al-Washlah, Al-Uraija, and Al-Muzahmiya populations was significantly shorter ($P < 0.05$) than that of the Al-Masanie population (Table 2).

The total-pre-oviposition period (TPOP) and adult-pre-oviposition period (APOP) of the Al-Masanie and Al-Washlah populations were significantly longer than those of the Dirab, Al-Uraija, and Al-Muzahmiya populations ($P < 0.05$). The oviposition days were significantly reduced in the Al-Washlah population compared with that of the Al-Muzahmiya population ($P < 0.05$), but similar with the other tested populations. The female ratio in the Al-Uraija population was significantly lower compared with the Dirab and Al-Washlah populations ($P < 0.05$). Whereas the reproductive female ratio was significantly lower in the Al-Washlah population compared with the other populations ($P < 0.05$). The fecundity/female was significantly lower in the Al-Washlah population than that of the Al-Masanie and Al-Muzahmiya populations ($P < 0.05$), but did not differ from that of the Dirab and Al-Uraija populations (Table 2).

Demographic life table features of house flies from dairies

The intrinsic rates of increase (r) in the Al-Masanie (0.013) and Al-Washlah (0.12) populations were significantly lower ($P < 0.05$) than that of the Dirab (0.17), Al-Uraija (0.18), and Al-Muzahmiya (0.19) populations. The finite rates of increase (λ) of the Al-Masanie and Al-Washlah (1.13) populations were significantly lower than that of the Dirab (1.18), Al-Uraija (1.19), and Al-Muzahmiya (1.21) populations ($P < 0.05$). The net reproductive rate (R_0) in the Al-Muzahmiya population (89.32) was significantly higher than that of the Al-Masanie (39.52) and Al-Washlah (33.04) populations ($P < 0.05$), and similar to that of the

Dirab (74.50) and Al-Uraija (64.35) populations. The generation time (T) in the Al-Masanie (29.43) and Al-Washlah (29.80) populations was significantly longer than that of the Dirab (26.05), Al-Uraija (23.54), and Al-Muzahmiya (24.08) populations ($P < 0.05$). The doubling time (DT) was shorter in the Al-Uraija (3.92) and Al-Muzahmiya (3.72) populations ($P < 0.05$) than that of the Al-Washlah population (5.91), but similar to that of the Dirab (4.19) and Al-Masanie (5.55) populations. Among all the tested populations, the GRR (gross reproduction rate) was similar ($P > 0.05$, Table 3).

Age-stage-specific survival rates (s_{xj}) of house flies from dairies

The parameter s_{xj} refers to the possible survival of newly born eggs to age x and development to stage j (Fig. 1). In all the populations the peak value of s_{xj} for larvae was similar. However, the highest peak values of s_{xj} for pupae in the Dirab (0.80) and Al-Washlah (0.70) populations were lower than those of the Al-Masanie (0.89), Ai-Uraija (0.99), and Al-Muzahmiya (0.97) populations. Similarly, the highest peak values of s_{xj} for females and males in the Dirab (0.25 and 0.24, respectively), Al-Uraija (0.21 and 0.49, respectively), and Al-Muzahmiya (0.29 and 0.36, respectively) populations were higher than those for the Al-Masanie (0.11 and 0.14, respectively) and Al-Washlah (0.19 and 0.13, respectively) populations.

Age-specific survival rates (l_x), fecundities (m_x, f_x), and maturity (l_xm_x) of house flies from dairies

The parameters l_x, f_x, m_x and l_xm_x were determined for all populations (Fig. 2). Among all populations, non-significant differences were found in the highest peak values for l_x. The highest peaks of f_x in the Dirab (40.67 eggs female^{-1}day^{-1}) and Al-Muzahmiya (40.06 eggs female^{-1}day^{-1}) populations were lower than those in the Al-Masanie (44.33 eggs female^{-1}day^{-1}), Al-Washlah (43.75 eggs female^{-1}day^{-1}), and Al-Uraija (55.67 eggs female^{-1}day^{-1}) populations, while the highest peaks of m_x in the Dirab (19 eggs individual^{-1}day^{-1}), Al-Masanie (20.71 eggs individual^{-1}day^{-1}), and Al-Uraija (21.97 eggs individual^{-1}day^{-1})

populations were lower than those in the Al-Washlah (25 eggs individual^{-1}day^{-1}) and Al-Muzahmiya (34.43 eggs individual^{-1}day^{-1}) populations. Additionally, the peak times of l_x, f_x and m_x for the Dirab (45th day), Al-Masanie (29th day), Al-Washlah (43th day), Al-Uraija (20th day), and Al-Muzahmiya (31th day) populations varied significantly among populations. Similarly, the highest peak values for l_xm_x in the Al-Masanie (4.35 at 24th day) and Al-Washlah (2.68 at 25th day) populations were lower than those in the Dirab (6.08 at 21th day), Al-Uraija (8.35 at 20th day), and Al-Muzahmiya (7.12 at 21th day) populations.

Age-stage-specific reproductive values (v_{xj}) of house flies from dairies

The v_{xj} reveals the reproductive value of insects at the age x and stage j in terms of future progeny (Fig. 3). The peak v_{xj} values for larvae were 6.54, 3.91, 4.11, 3.44, and 5.52 day^{-1} in the Dirab, Al-Masanie, Al-Washlah, Al-Uraija, and Al-Muzahmiya populations, respectively. The peak v_{xj} values for pupae in the Dirab (12.35 day^{-1}) Al-Masanie (7.49 day^{-1}), Al-Uraija (11.41 day^{-1}), and Al-Muzahmiya (11.56 day^{-1}) populations were lower than those in the Al-Washlah population (18.00 day^{-1}). The peak v_{xj} values for females in the Al-Masanie (166.7 day^{-1}), Al-Washlah (160.92 day^{-1}) and Al-Uraija (169.90 day^{-1}) populations were significantly higher than those in the Dirab (126.12 day^{-1}) and Al-Muzahmiya (132.47 day^{-1}) populations. The duration was shorter in the Al-Uraija and Al-Muzahmiya populations than that in the Dirab, Al-Masanie, and Al-Washlah populations.

Age-stage-specific life expectancy (e_{xj}) of house flies from dairies

The e_{xj} refers to the expected days over which an insect at age x and stage j will survive after age x shown in Fig. 4. The e_{xj} values for eggs were 22.13, 21.33, 16.48, 21.10, and 24.61 days in the Dirab, Al-Masanie, Al-Washlah, Al-Uraija, and Al-Muzahmiya populations, respectively. In the Al-Uraija and Al-Washlah populations, the peak e_{xj} values for larvae were 20.1 and 20.69 days, respectively, which were shorter than those in the Dirab (25.16 days), Al-Masanie (21.72), and Al-Muzahmiya (23.61 days) populations. The peak e_{xj} values for

pupae were 19.16, 14.72, 17.69, 16.1, and 18.76 days in the Dirab, Al-Masanie, Al-Washlah, Al-Uraija, and Al-Muzahmiya populations, and each of these values differed significantly. The peak e_{xj} values for the female and male house fly differed significantly in the tested populations—Al-Masanie (26.1 days and 24.73 days, respectively), Al-Washlah (24.88 days and 29.46 days, respectively), Al-Muzahmiya (21.60 days and 16.13 days, respectively), Dirab (19.80 days and 19.81 days, respectively), and Al-Uraija (18.80 days and 18.30 days, respectively).

Discussion

In the current study, the five populations of house fly collected from dairy facilities showed susceptibility to cyromazine, low resistance to methoxyfenozide and triflumuron, low–moderate resistance to pyriproxyfen and diflubenzuron in comparison to the susceptible strain. The insect populations that showed more than tenfold resistance to insecticides are known to be resistant [5, 49]. In this study, less than tenfold resistance to methoxyfenozide, cyromazine and triflumuron in all tested populations, pyriproxyfen in two populations, and diflubenzuron in one population were detected. These populations were considered tolerant rather than resistant. Previously, resistance to these insecticides has been found in house fly [20-22], *Phenacoccus solenopsis* Tinsley (Homoptera: Pseudococcidae) [50], *Spodoptera litura* (Fabri[illegible]s) (Lepidoptera: Noctuidae) [51], and *Spodoptera frugiperda* (J.E. Smith) (Lepidoptera: Noctuidae) [52]. However, this is the first susceptibility report of house fly populations to IGRs from dairy facilities in Riyadh, Saudi Arabia. Among the five tested IGRs, methoxyfenozide, cyromazine, and triflumuron were found to be the most toxic larvicides against house fly larvae. Therefore, these larvicides could be the most promising agents for the integrated vector management system in Saudi Arabia. However, the potential cross-resistance from other chemical classes [17] should be considered when aiming to design

a successful IGRs rotation pattern which requires further studies to prolong the effectiveness of these larvicides. Additionally, use of cultural practices (sanitation) and biological control agents with inclusion of insecticides for the control of house fly should be considered as an integrated pest management tool [12, 13].

The adaptation of an insect pest to an altered environment and insecticide applications depends on its life table parameters, and evidence defining its population dynamics is crucial in formulating an effective pest management plan. The present study was conducted on the five house fly populations from different dairy farms in Riyadh, Saudi Arabia, to explore their life table dynamics. Results revealed prominent variations in the larval duration, pupal duration, pre-adult male and female duration, oviposition days, longevity of males and females, APOP, TPOP, female ratio, and fecundity female^{-1}. The reason for the differences in life history features could be attributed to altered elevations, latitudes, and environmental factors—for instance, temperature and humidity—favoring the adaptation of the house flies. Similar to those in the current results, significant variations in life history parameters have been reported in several differing isolated pest populations [29-31, 53, 54]. For example, the developmental time of *P. xylostella* populations from higher latitudes was longer than that from lower latitudes [32]. In contrast, Shirai [54] reported no difference in the immature developmental time among nine geographically separate *P. xylostella* populations. The larval duration in *Colaphellus bowringi* Baly (Coleoptera: Chrysomelidae) was increased with increasing latitudes [30], but decreased in geometrid moths; *Cabera exanthemata* (Scopoli), *Lomaspilis marginata* (Linnaeus), *Chiasmia clathrata* (Linnaeus), and *Cabera pusaria* (Linnaeus) (Lepidoptera: Geometridae) [53]. Chen et al. [29] reported a higher net reproductive rate at 24 ˚C than at other tested temperatures in *P. crisonalis*. In house fly populations from Pakistan, the immature developmental time was shorter and pupal weights were heavier in those from lower latitudes with hot climates [31]. The aforementioned

variations among different insect pests could be due to the varied latitudes and favorable environments responsible for the flexibility of insect pests in their particular regions [30, 55], and/or the fitness costs accompanying with the development of insecticide resistance [1, 2, 56] due to pesticide application for the control of the house fly by dairy farmers.

The present results exhibited variations in the demographic parameters (r, λ, R_O, T, and DT) among the five house fly populations. The r and λ of the Al-Masanie and Al-Washlah populations were significantly lower than those in other tested field populations. The Al-Muzahmiya population exhibited the highest *Ro* among all of the field populations. The mean generation time of the Al-Uraija and Al-Muzahmiya populations was significantly shorter than that of other tested populations, while the doubling time of the Al-Washlah population was higher than those of the Al-Uraija and Al-Muzahmiya populations. The parameters r, λ, and R_O provide the growth potential estimation of pests—a wider insight than that provided by individual life history features [2]. Because the parameters r and λ depend upon the fecundity and growth of individuals, therefore differences in these parameters might affect the expansion rates of populations [2, 57, 58]. The lower rates of increase in the populations in this study could be attributable to lower fecundity female^{-1}. In agreement with present results, significant variations in demographic life table parameters of pests have previously been reported in *P. crisonalis* [29], *M. femurrubrum* [33], and *P. xylostella* [32],

The parameters s_{xj}, l_x, f_x, m_x, l_xm_x, $v_{xj,}$ and e_{xj} are important indicators for evaluating the biological fitness of insect pest populations. Similar insect pests under altered environments have different survival rates, reproduction abilities, and life spans, all of which reflect specific environmental effects and species-specific parameters [59, 60]. The present results for s_{xj}, l_x, f_x, m_x, l_xm_x, $v_{xj,}$ and e_{xj} revealed significant variations in house fly populations from five different dairy farms. Because of differing environmental factors—for instance, temperature—and extents of insecticide exposure, significant variations in these

parameters have previously been documented in a range of pests, including *P. crisonalis* [29], *Sogatella furcifera* (Horvath) (Hemiptera: Delphacidae) [60], *Bradysia odoriphaga* Yang and Zhang (Diptera: Sciaridae) [42], *Phthorimaea operculella* Zeller (Lepidoptera: Gelechiidae) [61], *S. litura* [27], and *Tetranychus urticae* Koch (Acari: Tetranychidae) [62].

Effectiveness and implementation of control measures against insect pests depend on knowledge of the life table attributes of any pest in its respective environment [31, 32]. Our results provide useful insights about the age, stage, and two sexes based life table variation in house fly populations from Riyadh, Saudi Arabia. The higher *r* and λ values and shorter T values in the Dirab, Al-Uraija, and Al-Muzahmiya populations revealed similarities among these populations, while they differed significantly from the Al-Masanie and Al-Washlah populations. In the house fly, a shorter life span predicts the development of insecticide resistance; therefore present data might be helpful in predicting the growth rates of house fly populations in geographically separated dairy farms and in formulating timely management strategies for this pest in specific dairies.

Acknowledgments

The authors extend their appreciation to the Deputyship for Research & Innovation, “Ministry of Education” in Saudi Arabia for funding this research work through the project number IFKSURG-1441-480. The authors thank the Deanship of Scientific Research and Researchers Support and Services Unit (RSSU) at King Saud University for their technical support. Authors would like also to thank the researchers and technicians from Pesticides and Environmental Toxicology Laboratory: Ahmed Mohammed Dabo and Safwat Gamal Sabra for their help in collecting and maintaining the house fly field populations in the laboratory and for other laboratory work.

Conflict of interest

The authors have declared no conflict of interest.

Data Availability Statement

All relevant data are within the manuscript and its Supporting Information files.

References

1. Abbas N, Shah RM, Shad SA, Azher F. Dominant fitness costs of resistance to fipronil in *Musca domestica* Linnaeus (Diptera: Muscidae). Vet Parasitol. 2016;226:78-82.
2. Shah RM, Shad SA, Abbas N. Methoxyfenozide resistance of the housefly, *Musca domestica* L. (Diptera: Muscidae): cross-resistance patterns, stability and associated fitness costs. Pest Manag Sc[illegible]017;73(1):254-61.
3. Fasanella A, Scasciamacchia S, Garofolo G, Giangaspero A, Tarsitano E, Adone R. Evaluation of the house fly *Musca domestica* as a mechanical vector for an anthrax. Plos One. 2010;5(8):e12219.
4. Förster M, Klimpel S, Mehlhorn H, Sievert K, Messler S, Pfeffer K. Pilot study on synanthropic flies (e.g. *Musca*, *Sarcophaga*, *Calliphora*, *Fannia*, *Lucilia*, *Stomoxys*) as vectors of pathogenic microorganisms. Parasitol Res. 2007;101(1):243-6.
5. Abbas N, Shad SA, Ismail M. Resistance to conventional and new insecticides in house flies (Diptera: Muscidae) from poultry facilities in Punjab, Pakistan. J Econ Entomol. 2015;108(2):826–33.
6. Abbas N, Ijaz M, Shad SA, Binyameen M. Assessment of resistance risk to fipronil and cross resistance to other insecticides in the *Musca domestica* L. (Diptera: Muscidae). Vet Parasitol. 2016;223:71-6.
7. Khan HAA, Akram W, Fatima A. Resistance to pyrethroid insecticides in house flies, *Musca domestica* L., (Diptera: Muscidae) collected from urban areas in Punjab, Pakistan. Parasitol Res. 2017;116(12):3381-5.
8. Freeman JC, Ross DH, Scott JG. Insecticide resistance monitoring of house fly populations from the United States. Pestic Biochem Physiol. 2019;158:61-8.
9. Li Q, Huang J, Yuan J. Status and preliminary mechanism of resistance to insecticides in a field strain of house fly (*Musca domestica*, L). Rev Bras Entomol. 2018;62(4):311-4.
10. Shah RM, Shad SA. House fly resistance to chlorantraniliprole: cross resistance patterns, stability and associated fitness costs. Pest Manag Sci. 2020;76(5):1866-73.
11. Abbas N, Khan HAA, Shad SA. Resistance of the house fly *Musca domestica* (Diptera: Muscidae) to lambda-cyhalothrin: mode of inheritance, realized heritability, and cross-resistance to other insecticides. Ecotoxicology. 2014;23(5):791-801.
12. Malik A, Singh N, Satya S. House fly (*Musca domestica*): a review of control strategies for a challenging pest. Journal of Environmental Science and Health Part B[illegible] 2007;42(4):453-69.
13. Farooq M, Freed S. Infectivity of housefly, *Musca domestica* (Diptera: Muscidae) to different entomopathogenic fungi. Braz J Microbiol. 2016;47(4):807-16.
14. Tunaz H, Uygun N. Insect growth regulators for insect pest control. Turkish Journal [illegible] Agriculture and Forestry. 2004;28(6):377-87.
15. Mian LS, Mulla MS. Biological and environmental dynamics of insect growth regulators (IGRs) as used against Diptera of public health importance. Residue Reviews: [illegible] Springer; 1982. p. 27-112.

16. Kočišová A, Petrovský M, Toporčák J, Novák P. The pote[illegible]l of some insect growth regulators in housefly (*Musca domestica*) control. Biologia, Bratisl[illegible] 2004;59(5):661-8.
17. IRAC. IRAC: Mode of action classification scheme, Version 9.4, .www.irac-online.org › documents › moa-classification. 2020:pp, 1-26.
18. Shah RM, Abbas N, Shad SA, Sial AA. Selection, resistance risk assessment, and reversion toward susceptibility of pyriproxyfen in *Musca domestica* L. Parasitol Res. 2015;114(2):487-94.
19. Matsumura F. Studies on the action mechanism of benzoylurea insecticides to inhibit the process of chitin synthesis in insects: A review on the status of research activities in the past, the present and the future prospects. Pestic Biochem Physiol. 2010;97(2):133-9.
20. Abbas N, Shad SA, Shah RM. Resistance status of *Musca domestica* L. populations to neonicotinoids and insect growth regulators in Pakistan poultry facilities. Pak J Zool. 2015;47(6):1663-71.
21. Khan HAA, Akram W, Arshad M, Hafeez F. Toxicity and resistance of field collected *Musca domestica* (Diptera: Muscidae) against insect growth regulator insecticides. Parasitol Res. 2016;115(4):1385-90.
22. Bell HA, Robinson KA, Weaver RJ. First report of cyromazine resistance in a population of UK house fly (*Musca domestica*) associated with intensive livestock production. Pest Manag Sci. 2010;66(7):693-5.
23. Tang JD, Caprio MA, Sheppard DC, Gaydon DM. Genetics and fitness costs of cyromazine resistance in the house fly (Diptera: Muscidae). J Econ Entomol. 2002;95(6):1251-60.
24. Shah RM, Shad SA, Abbas N. Mechanism, stability and fitness cost of resistance to pyriproxyfen in the house fly, *Musca domestica* L. (Diptera: Muscidae). Pestic Biochem Physiol. 2015;119:67-73.
25. Shah RM, Abbas N, Shad SA, Binyameen M. Determination of the genetic and synergistic suppression of a methoxyfenozide-resistant strain of the house fly *Musca domestica* L. (Diptera: Muscidae). Neotrop Entomol. 2018;47(5):709-15.
26. Chi H, You M, Atlıhan R, Smith CL, Kavousi A, Özgökçe MS, et al. Age-stage, two-sex life table: an introduction to theory, data analysis, and application. Entomol Gen. 2020;40(2):102-23.
27. Tuan SJ, Lee CC, Chi H. Population and damage projection of *Spodoptera litura* (F.) on peanuts (*Arachis hypogaea* L.) under different conditions using the age-stage, two-sex life table. Pest Manag Sci. 2014;70(5):805-13.
28. Chi H. Life-table analysis incorporating both sexes and variable development rates among individuals. Environ Entomol. 1988;17(1):26-34.
29. Chen Q, Li N, Wang X, Ma L, Huang J-B, Huang G-H. Age-stage, two-sex life table of *Parapoynx crisonalis* (Lepidoptera: Pyralidae) at different temperatures. PloS one. 2017;12(3):e0173380.
30. Tang J, He H, Chen C, Fu S, Xue F. Latitudinal cogradient variation of development time and growth rate [illegible] a negative latitudinal body weight cline in a widely distributed cabbage beetle. PloS o[illegible]2017;12(7):e0181030.
31. Khan HAA, Khan MU, Nasiba A, Riaz S, Altaf M. Geographical varia[illegible]s in life histories of house flies, *Musca domestica* (Diptera: Muscidae), in Punjab, Paki[illegible]. J Med Entomol. 2019;56(5):1225-30.
32. Pan Q-J, Chen L, Lin X-L, Ridsdill-Smith TJ, Liu T-X. Geographical variations in life histories of *Plutella xylostella* in China. J Pest Sci. 2014;87(4):659-70.
33. Parsons SMA, Joern A. Life history traits associated with body size covary along a latitudinal gradient in a generalist grasshopper. Oecologia. 2014;174(2):379-91.

34. Saeed R, Abbas N, Razaq M, Mahmood Z, Naveed M, Rehman HMU. Field evolved resistance to pyrethroids, neonicotinoids and biopesticides in *Dysdercus koenigii* (Hemiptera: Pyrrhocoridae) from Punjab, Pakistan. Chemosphere. 2018;213:149-55. doi: 10.1016/j.chemosphere.2018.09.042. PubMed PMID: WOS:000448493700016.
35. Saeed R, Razaq M, Abbas N, Jan MT, Naveed M. Toxicity and resistance of the cotton leaf hopper, *Amrasca devastans* (Distant) to neonicotinoid insecticides in Punjab, Pakistan. Crop Prot. 2017;93:143-7.
36. Ullah S, Shad SA, Abbas N. Resistance of dusky Cotton bug, *Oxycarenus hyalinipennis* Costa (Lygaidae: Hemiptera), to conventional and novel chemistry insecticides. J Econ Entomol. 2016;109(1):345-51.
37. Khan HAA, Akram W, Lee S. Resistance to selected pyrethroid insecticides in the malaria mosquito, *Anopheles stephensi* (Diptera: Culicidae), from Punjab, Pakistan. J Med Entomol. 2018;55(3):735-8.
38. Kaufman PE, Nunez SC, Mann RS, Geden CJ, Scharf ME. Nicotinoid and pyrethroid insecticide resistance in houseflies (Diptera: Muscidae) collected from Florida dairies. Pest Manag Sci. 2010;66(3):290-4.
39. Wang J-N, Hou J, Wu Y-Y, Guo S, Liu Q-M, Li T-Q, et al. Resistance of house fly, *Musca domestica* L. (Diptera: Muscidae), to five insecticides in Zhejiang Province, China: The situation in 2017. Canadian Journal of Infectious Diseases and Medical Microbiology. 2019;2019:1-10.
40. Khan HAA, Akram W, Shad SA. Resistance to conventional insecticides in Pakistani populations of *Musca domestica* L. (Diptera: Muscidae): a potential ectoparasite of dairy animals. Ecotoxicology. 2013;22:522-7.
41. Abbas N, Khan HAA, Shad SA. Cross-resistance, genetics, and realized heritability of resistance to fipronil in the house fly, *Musca domestica* (Diptera: Muscidae): a potential vector for disease transmission. Parasitol Res. 2014;113(4):1343–52.
42. Gul H, Ullah F, Biondi A, Desneux N, Qian D, Gao X, et al. Resistance against clothianidin and associated fitness costs in the chive maggot, *Bradysia odoriphaga*. Entomol Gen. 2019;39(2):81-92.
43. Chi H, Su H-Y. Age-stage, two-sex life tables of *Aphidius gifuensis* (Ashmead) (Hymenoptera: Braconidae) and its host *Myzus persicae* (Sulzer) (Homoptera: Aphididae) with mathematical proof of the relationship between female fecundity and the net reproductive rate. Environ Entomol. 2006;35(1):10-21.
44. LeOra S. Poloplus, a user's guide to probit or logit analysis. LeOra Software, Berkeley, CA. 2003.
45. Abbott W. A method of computing the effectiveness of an insecticide. J Econ Entomol. 1925;18(2):265-7.
46. Ahmad M, Iqbal Arif M, Ahmad M. Occurrence of insecticide resistance in field populations of *Spodoptera litura* (Lepidoptera: Noctuidae) in Pakistan. Crop Prot. 2007;26(6):809-17.
47. Chi H. TWOSEX-MSChart: a computer program for the age-stage, two-sex life table analysis. http://140120197173/Ecology/prod02htm. 2019.
48. Chi H, Yang T-C. Two-sex life table and predation rate of *Propylaea japonica* Thunberg (Coleoptera: Coccinellidae) fed on *Myzus persicae* (Sulzer) (Homoptera: Aphididae). Environ Entomol. 2003;32(2):327-33.
49. Valles SM, Koehler PG, Brenner RJ. Antagonism of fipronil toxicity by piperonyl butoxide and S, S, S-tributyl phosphorotrithioate in the German cockroach (Dictyoptera: Blattellidae). J Econ Entomol. 1997;90(5):1254-8.

50. Saddiq B, Shad SA, Aslam M, Ijaz M, Abbas N. Monitoring resistance of *Phenacoccus solenopsis* Tinsley (Homoptera: Pseudococcidae) to new chemical insecticides in Punjab, Pakistan. Crop Prot. 2015;74:24-9.

51. Ahmad M, Gull S. Susceptibility of armyworm *Spodoptera litura* (Lepidoptera: Noctuidae) to novel insecticides in Pakistan. The Canadian Entomologis[illegible]017;149(5):649-61.

52. Gutiérrez-Moreno R, Mota-Sanchez D, Blanco CA, Whalon ME, Terán-Santofimio H, Rodriguez-Maciel JC, et al. Field-evolved resistance of the fall armyworm (Lepidoptera: Noctuidae) to synthetic insecticides in Puerto Rico and Mexico. J Econ Entomol. 2019;112(2):792-802.

53. Kivelä SM, Välimäki P, Carrasco D, Mäenpää MI, Oksanen J. Latitudinal insect body size clines revisited: a critical evaluation of the saw-tooth model. J Anim Ecol. 2011;80(6):1184-95.

54. Shirai Y. Temperature tolerance of the diamondback moth, *Plutella xylostella* (Lepidoptera: Yponomeutidae) in tropical and temperate regions of Asia. Bull Entomol Res. 2000;90(4):357-64.

55. Honek A. Geographical variation in thermal requirements for insect development. Eur J Entomol. 2013;93(3):303-12.

56. Abbas N, Shah RM, Shad SA, Iqbal N, Razaq M. Biological trait analysis and stability of lambda-cyhalothrin resistance in the house fly, *Musca domestica* L. (Diptera: Muscidae). Parasitol Res. 2016;115(5):2073-80.

57. Abbas N, Khan H, Shad SA. Cross-resistance, stability, and fitness cost of resistance to imidacloprid in *Musca domestica* L., (Diptera: Muscidae). Parasitol Res. 2015;114(1):247-55. Epub 2014/10/25. PubMed PMID: 25342464.

58. Abbas N, Shad SA, Razaq M. Fitness cost, cross resistance and realized heritability of resistance to imidacloprid in *Spodoptera litura* (Lepidoptera: Noctuidae). Pestic Biochem Physiol. 2012;103(3):181-8.

59. Zhang P, Zhao Y-H, Wang Q-H, Mu W, Liu F. Lethal and sublethal effects of the chitin synthesis inhibitor chlorfluazuron on *Bradysia odoriphaga* Yang and Zhang (Diptera: Sciaridae). Pestic Biochem Physiol. 2017;136:80-8.

60. Xiang X, Liu S, Wang X, Zhang Y, Gong C, Chen L, et al. Sublethal effects of sulfoxaflor on population projection and development of the white-backed planthopper, *Sogatella furcifera* (Hemiptera: Delphacidae). Crop Prot. 2019;120:97-102.

61. Yuan H-g, Wu S-y, Lei Z-r, Rondon SI, Gao Y-l. Sub-lethal effects of *Beauveria bassiana* (Balsamo) on field populations of the potato tuberworm *Phthorimaea operculella* Zeller in China. Journal of Integrative Agricultur[illegible]018;17(4):911-8.

62. Wang L, Zhang Y, Xie W, Wu Q, Wang S. Sublethal effects of spinetoram on the two-spotted spider mite, *Tetranychus urticae* (Acari: Tetranychidae). Pestic Biochem Physiol. 2016;132:102-7.

Table 1 Resistance to insect growth regulators in the larvae of different dairy farms *M. domestica* populations

Insecticide	Population	Year	[1]N	Slope ± SE	χ^2 (df)	P	[2]LC_{50}	[3]FL (95%)	[4]RR
Pyriproxyfen	Susceptible (G_{14})	2019	240	1.52 ± 0.19	6.17 (3)	0.10	0.04	0.03–0.06	1.00
	Dirab (G_1)	2019	240	1.63 ± 0.27	5.07 (3)	0.17	1.19	0.26–2.38	29.75
	Al-Masanie (G_1)	2019	240	2.21 ± 0.34	5.98 (3)	0.11	0.70	0.16–1.19	17.50
	Al-Uraija (G_1)	2019	240	1.68 ± 0.32	1.52 (3)	0.68	0.43	0.21–0.63	10.75
	Al-Washlah (G_1)	2019	240	0.80 ± 0.24	1.00 (3)	0.80	0.22	0.01–0.53	5.50
	Al-Muzahmiya (G_1)	2019	240	1.31 ± 0.29	0.77 (3)	0.86	0.33	0.10–0.56	8.25
Diflubenzuron	Susceptible (G_{14})	2019	240	1.49 ± 0.34	2.27 (3)	0.52	0.03	0.01–0.05	1.00
	Dirab (G_1)	2019	240	1.87 ± 0.28	3.75 (3)	0.29	0.41	0.19–0.70	13.67
	Al-Masanie (G_1)	2019	240	1.68 ± 0.24	3.17 (3)	0.37	0.46	0.28–0.74	15.33
	Al-Uraija (G_1)	2019	240	1.04 ± 0.24	0.10 (3)	0.99	0.28	0.10–0.46	9.33
	Al-Washlah (G_1)	2019	240	1.31 ± 0.17	4.45 (3)	0.22	0.86	0.55–1.40	28.67
	Al-Muzahmiya (G_1)	2019	240	1.53 ± 0.24	5.06 (3)	0.17	0.86	0.37–1.78	28.67
Triflumuron	Susceptible(G_{14})	2019	240	0.99 ± 0.32	0.13 (3)	0.99	0.08	0.00–0.26	1.00
	Dirab (G_1)	2019	240	2.24 ± 0.66	0.24 (3)	0.97	0.23	0.04–0.39	2.88
	Al-Masanie (G_1)	2019	240	1.08 ± 0.25	6.05 (3)	0.11	0.62	0.27–3.53	7.75
	Al-Uraija (G_1)	2019	240	1.83 ± 0.30	0.13 (3)	0.99	0.66	0.42–0.89	8.25
	Al-Washlah (G_1)	2019	240	1.67 ± 0.38	0.84 (3)	0.84	0.27	0.08–0.41	3.38
	Al-Muzahmiya (G_1)	2019	240	1.54 ± 0.47	1.03 (3)	0.79	0.13	0.01–0.30	1.63
Cyromazine	Susceptible (G_{14})	2019	240	2.54 ± 0.31	4.76 (3)	0.19	0.22	0.18–0.28	1.00
	Dirab (G_1)	2019	240	2.15 ± 0.27	1.57 (3)	0.67	0.16	0.13–0.21	0.73
	Al-Masanie (G_1)	2019	240	1.82 ± 0.36	4.76 (3)	0.19	0.64	0.43–1.34	2.91
	Al-Uraija (G_1)	2019	240	2.76 ± 0.39	1.30 (3)	0.73	0.29	0.24–0.37	1.32
	Al-Washlah (G_1)	2019	240	2.47 ± 0.32	6.88 (3)	0.08	0.22	0.13–0.40	1.00
	Al-Muzahmiya (G_1)	2019	240	3.43 ± 0.39	6.09 (3)	0.12	0.13	0.11–0.15	0.59
Methoxyfenozide	Susceptible (G_{14})	2019	240	2.13 ± 0.26	5.38 (3)	0.15	4.99	3.91–6.40	1.00
	Dirab (G_1)	2019	240	1.58 ± 0.36	3.26 (3)	0.35	34.55	23.27–80.48	6.92
	Al-Masanie (G_1)	2019	240	3.09 ± 0.45	1.34 (3)	0.72	40.09	33.46–50.03	8.03
	Al-Uraija (G_1)	2019	240	3.25 ± 0.38	0.94 (3)	0.82	21.55	18.25–25.58	4.32
	Al-Washlah (G_1)	2019	240	2.15 ± 0.27	1.57 (3)	0.67	20.79	16.75–26.30	4.17
	Al-Muzahmiya (G_1)	2019	240	1.56 ± 0.24	0.59 (3)	0.90	18.79	14.16–25.48	3.77

[1] Number of tested larvae. [2] Median lethal concentrations in µg/ml. [3] Fiducial limits [4] Resistance ratios.

Table 2 Development duration and life history parameters of five house fly populations collected from dairies in Riyadh, Saudi Arabia

Parameters	N	Dirab ±SEM*	N	Al-Masanie ±SEM*	N	Al-Washlah ±SEM*	N	Al-Uraija ±SEM*	N	Al-Muzahmiya ±SEM*
larvae (d)	80	7.89 ± 0.05 b	89	8.09 ± 0.03 a	70	6.80 ± 0.11 d	100	5.90 ± 0.04 e	100	7.34 ± 0.11 c
pupae (d)	50	5.68 ± 0.09 c	27	6.00 ± 0.11 b	33	5.58 ± 0.12 c	70	6.80 ± 0.06 a	65	5.11 ± 0.14 d
pre-adult ♂ (d)	25	14.60 ± 0.14 ab	16	15.00 ± 0.18 a	13	14.00 ± 0.39 bc	49	13.69 ± 0.11 c	36	12.89 ± 0.28 d
pre-adult ♀ (d)	25	14.56 ± 0.13 b	11	15.00 ± 0.00 a	20	14.35 ± 0.21 b	21	13.57 ± 0.27 c	29	13.35 ± 0.19 c
longevity ♂ (d)	25	32.84 ± 2.37 b	16	33.88 ± 3.56 ab	13	40.46 ± 2.49 a	49	22.57 ± 1.31 c	36	26.28 ± 1.66 c
longevity ♀ (d)	25	32.80 ± 2.19 ab	11	39.73 ± 3.20 a	20	27.00 ± 3.49 b	21	27.81 ± 2.01 b	29	30.52 ± 2.39 b
APOP[a] (d)	19	5.47 ± 0.41 b	10	8.70 ± 0.79 a	9	8.75 ± 0.93 a	15	4.80 ± 0.61 b	20	4.45 ± 0.42 b
TPOP[b] (d)	19	19.89 ± 0.42 b	10	23.70 ± 0.79 a	9	22.75 ± 0.58 a	15	18.20 ± 0.76 c	20	17.50 ± 0.45 c
oviposition (d)	25	6.42 ± 1.12 ab	11	6.90 ± 1.30 ab	20	5.88 ± 1.03 b	21	7.07 ± 0.70 ab	29	8.95 ± 0.93 a
female ratio	100	0.50 ± 0.007 a	100	0.41 ± 0.01 ab	100	0.61 ± 0.009 a	100	0.30 ± 0.005 b	100	0.45 ± 0.006 ab
rep. female ratio	100	0.76 ± 0.009 a	100	0.91 ± 0.009 a	100	0.40 ± 0.11 b	100	0.71 ± 0.10 a	100	0.69 ± 0.009 a
fecundity/female	25	298.00 ± 69.70 ab	11	359.27 ± 77.79 a	20	165.2 ± 52.32 b	21	306.43 ± 59.48 ab	29	308.00 ± 52.70 a

Different letters in each row indicate significant differences among the populations (paired bootstrap test, $P \leq 0.05$) using TWO-SEX MS chart program.

* Standard errors of means (SEM) were estimated using bootstrapping (100,000 re-samplings).

N = cohort size, d = days, rep. = reproductive, [a]adult pre-oviposition period, [b]total pre-oviposition period

Table 3 Demographic life table features of five house fly populations collected from dairies in Riyadh, Saudi Arabia

Parameters	Dirab ±SEM*	Al-Masanie ±SEM*	Al-Washlah ±SEM*	Al-Uraija ±SEM*	Al-Muzahmiya ±SEM*
intrinsic rate of increase (r) (d^{-1})	0.17 ± 0.01 a	0.13 ± 0.01 b	0.12 ± 0.02 b	0.18 ± 0.01 a	0.19 ± 0.01 a
finite rate of increase (λ) (d^{-1})	1.18 ± 0.01 a	1.13 ± 0.02 b	1.13 ± 0.02 b	1.19 ± 0.02 a	1.21 ± 0.01 a
net reproductive rate (R_0) (offspring/individual)	74.50 ± 21.45 ab	39.52 ± 13.88 b	33.04 ± 12.15 b	64.35 ± 17.40 ab	89.32 ± 20.56 a
mean generation time (T) (d)	26.05 ± 0.48 b	29.43 ± 0.89 a	29.80 ± 1.48 a	23.54 ± 0.47 c	24.08 ± 0.49 c
doubling time (DT) (d)	4.19 ± 0.34 ab	5.55 ± 1.07 ab	5.91 ± 1.06 a	3.92 ± 0.31 b	3.72 ± 0.24 b
gross reproduction rate (GRR) (offspring/individual)	305.78 ± 82.98 a	250.15 ± 79.20 a	240.46 ± 75.03 a	252.04 ± 64.02 a	344.18 ± 53.57 a

Different letters in each row indicate significant differences among the populations (paired bootstrap test, $P \leq 0.05$) using TWO-SEX MS chart program. *Standard errors of means (SEM) were estimated using bootstrapping (100,000 re-samplings). d = days

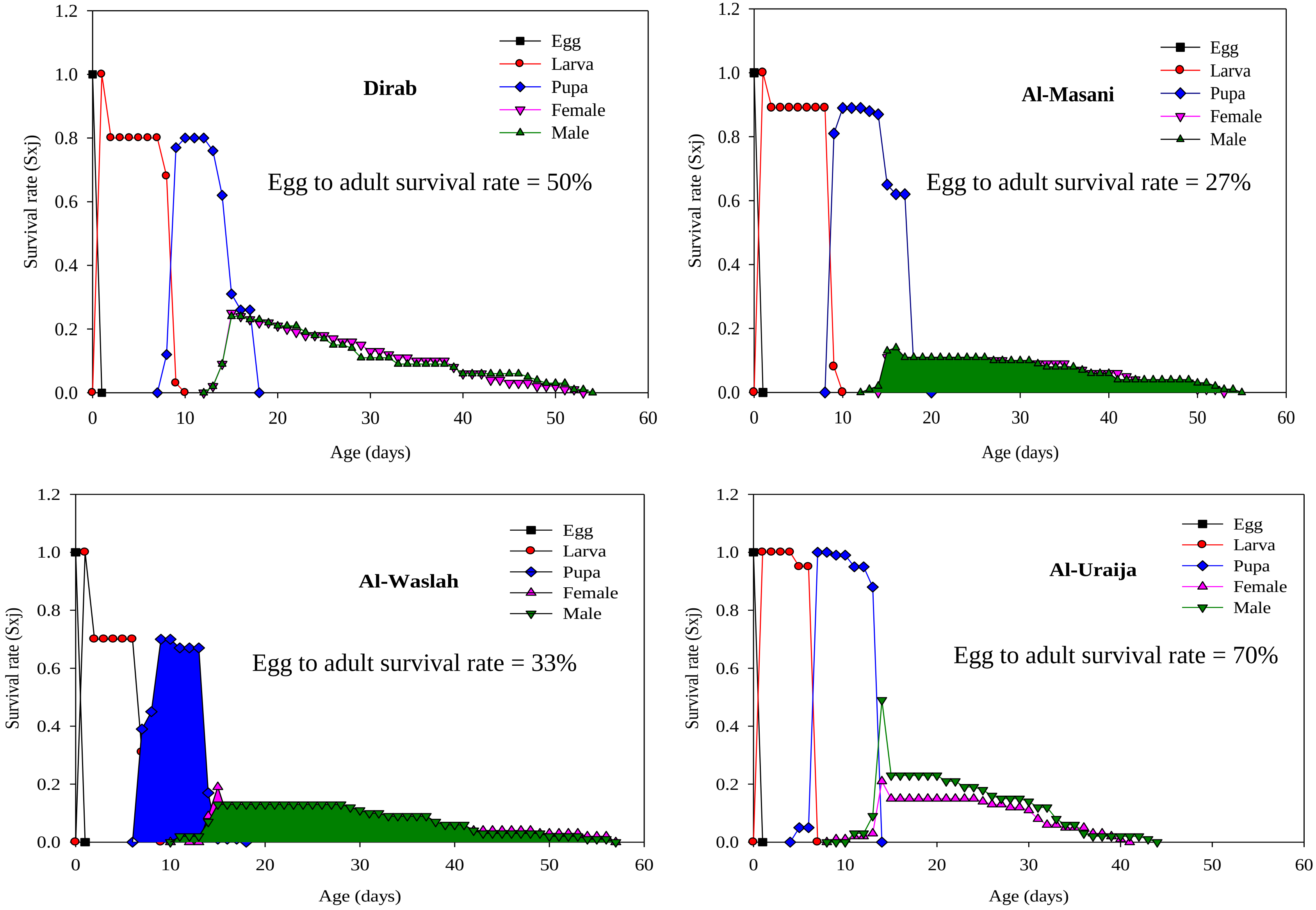
Dirab
Egg
Larva
Pupa
Female
Male
Egg to adult survival rate = 50%
Survival rate (Sxj)
Age (days)
Al-Masani
Egg
Larva
Pupa
Female
Male
Egg to adult survival rate = 27%
Survival rate (Sxj)
Age (days)
Al-Waslah
Egg
Larva
Pupa
Female
Male
Egg to adult survival rate = 33%
Survival rate (Sxj)
Age (days)
Al-Uraija
Egg
Larva
Pupa
Female
Male
Egg to adult survival rate = 70%
Survival rate (Sxj)
Age (days)

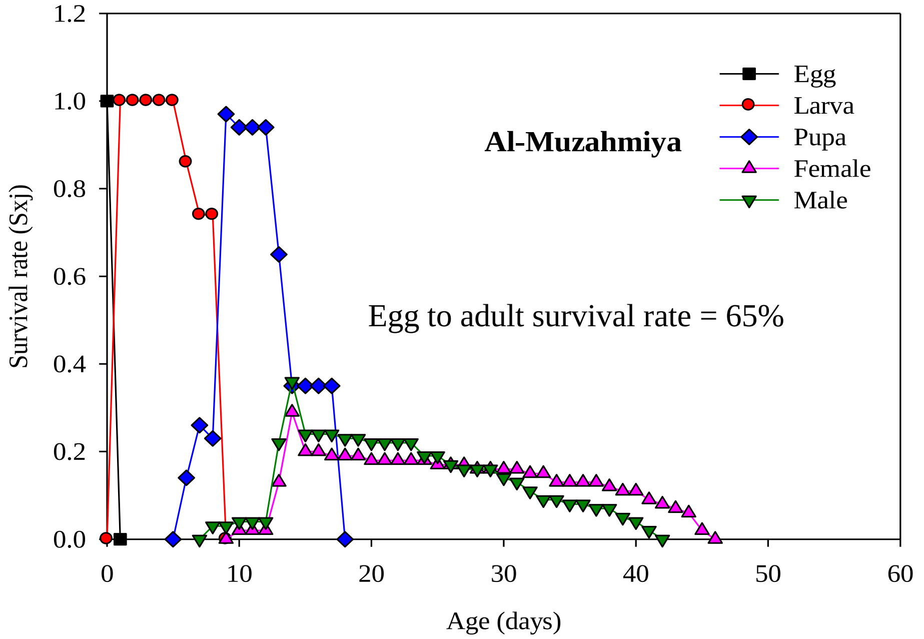

Fig. 1 Age-stage-specific survival rate (s_{xj}) for the Dirab, Al-Masanie, Al-Washlah, Al-Uraija, and Al-Muzahmiya populations of house fly.

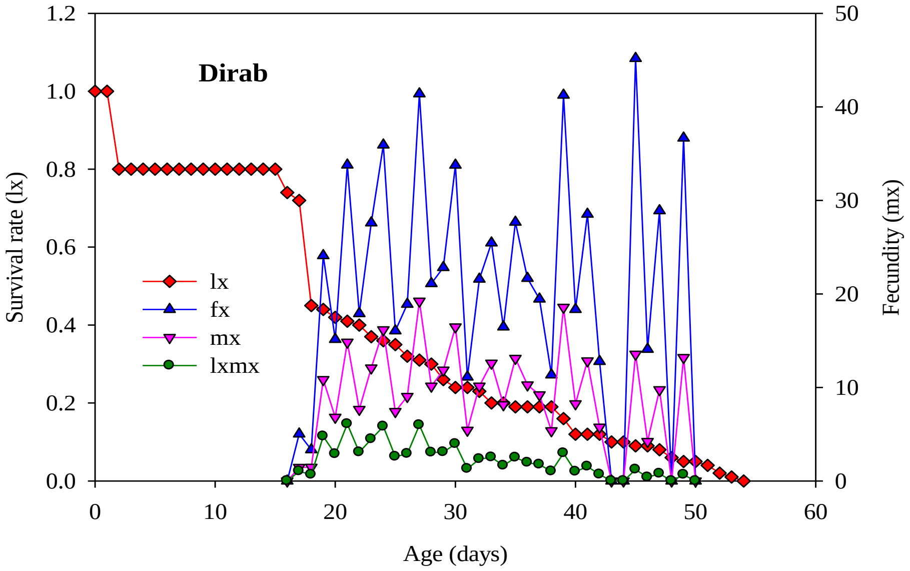
Dirab
Survival rate (lx)
Fecundity (mx)
Age (days)
lx
fx
mx
lxmx
1.2
1.0
0.8
0.6
0.4
0.2
0.0
50
40
30
20
10
0
0
10
20
30
40
50
60

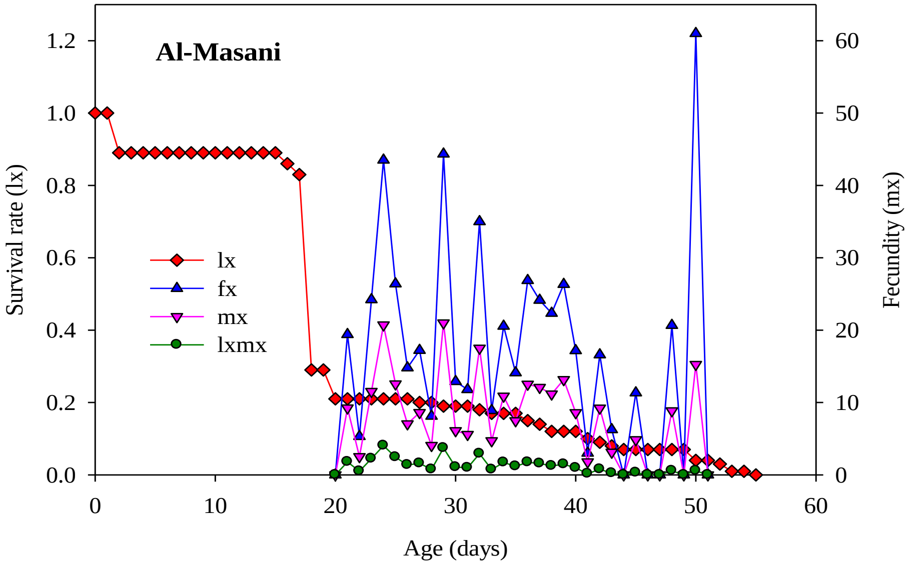
Al-Masani
Survival rate (lx)
Fecundity (mx)
Age (days)
lx
fx
mx
lxmx
1.2
1.0
0.8
0.6
0.4
0.2
0.0
60
50
40
30
20
10
0
0
10
20
30
40
50
60

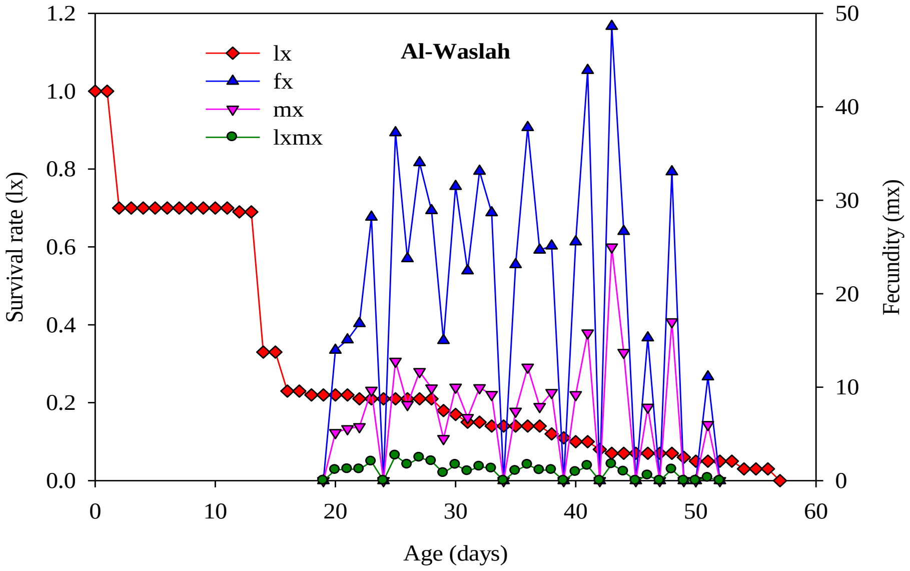
Al-Waslah
lx
fx
mx
lxmx
Survival rate (lx)
Fecundity (mx)
Age (days)
1.2
1.0
0.8
0.6
0.4
0.2
0.0
50
40
30
20
10
0
0
10
20
30
40
50
60

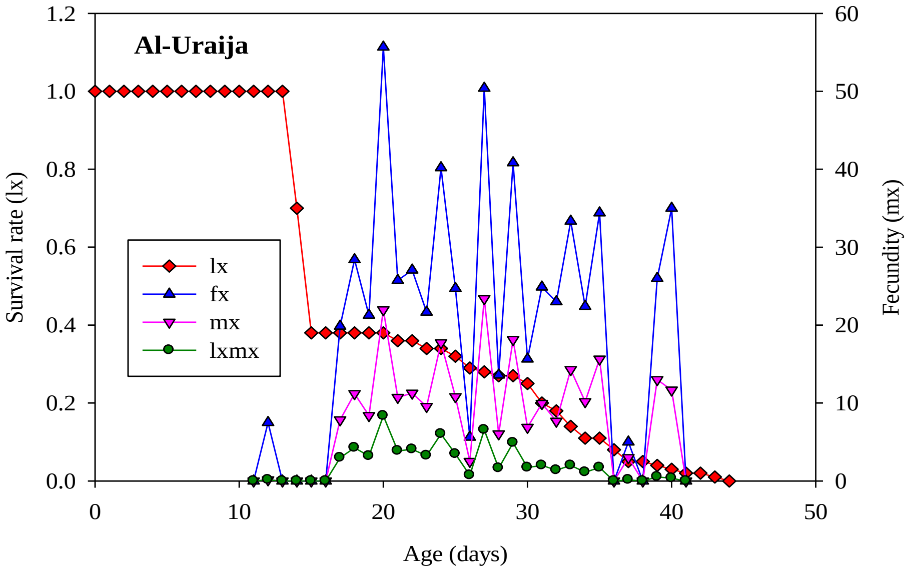
Al-Uraija
lx
fx
mx
lxmx
Survival rate (lx)
Fecundity (mx)
Age (days)
1.2
1.0
0.8
0.6
0.4
0.2
0.0
60
50
40
30
20
10
0
0
10
20
30
40
50

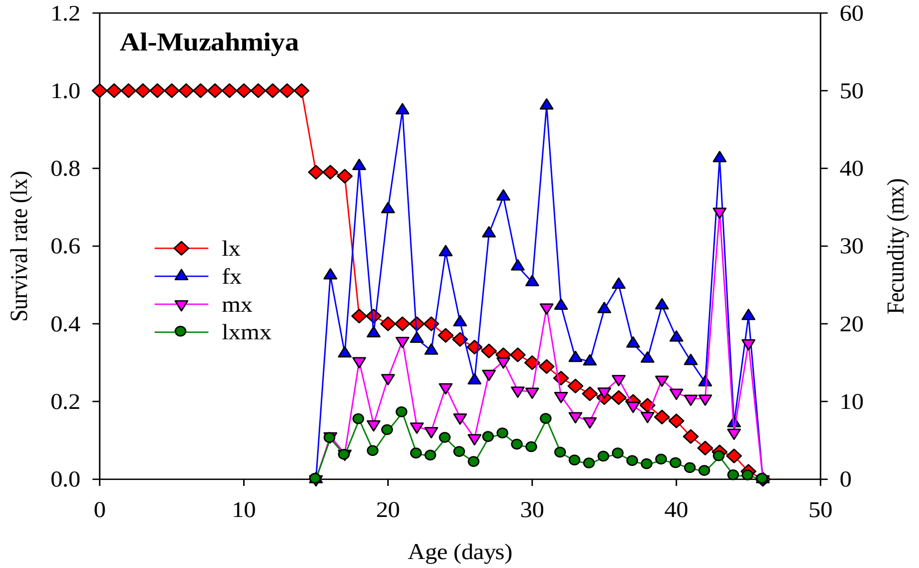

Fig. 2 Age-specific survival rate (l_x), age-specific female fecundity (f_x), age-specific fecundity of the total population (m_x), and age-specific maturity ($l_x m_x$) for the Dirab, Al-Masanie, Al-Washlah, Al-Uraija, and Al-Muzahmiya populations of house fly.

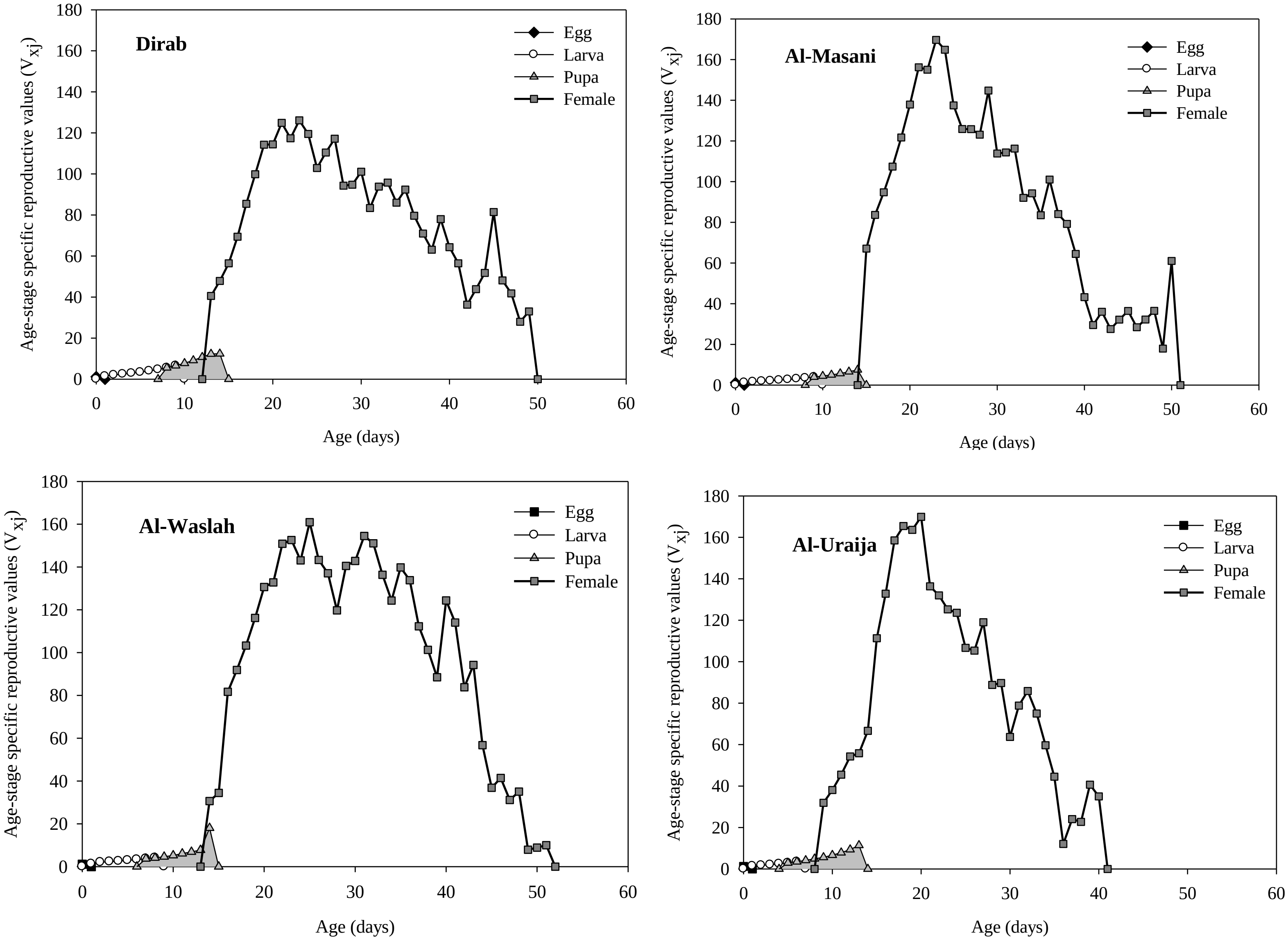

Dirab
Al-Masani
Al-Waslah
Al-Uraija
Egg
Larva
Pupa
Female
Age-stage specific reproductive values (V_{xj})
Age (days)

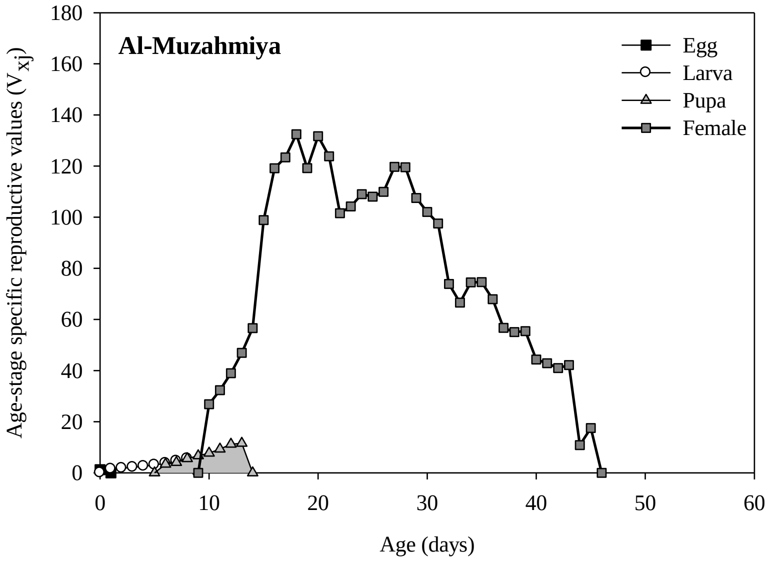

Fig. 3 Age-stage-specific reproductive values (v_{xj}) for the Dirab, Al-Masanie, Al-Washlah, Al-Uraija, and Al-Muzahmiya populations of house fly.

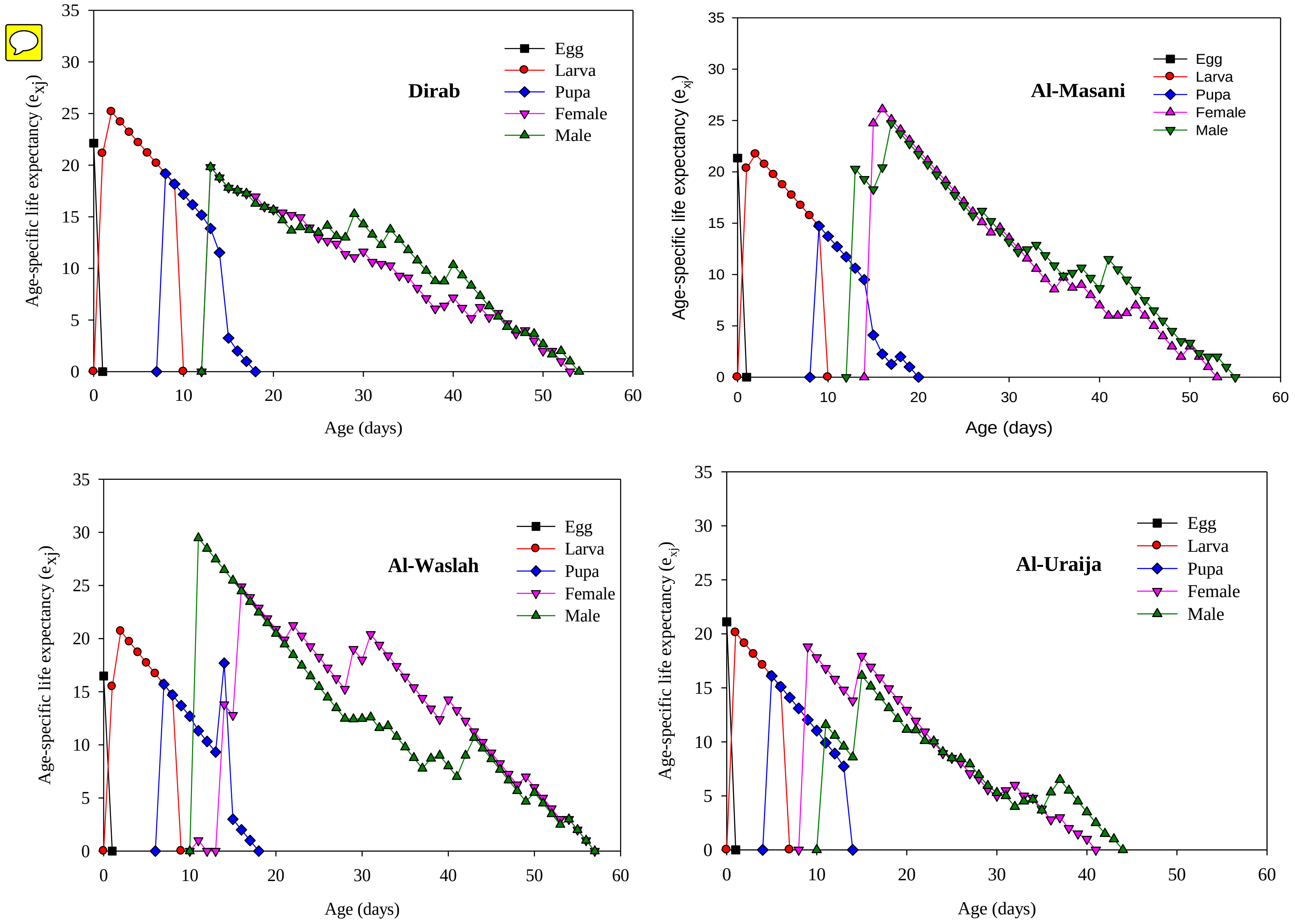
Dirab
Al-Masani
Al-Waslah
Al-Uraija
Egg
Larva
Pupa
Female
Male
Age-specific life expectancy (e_{xj})
Age (days)
0
5
10
15
20
25
30
35
40
50
60

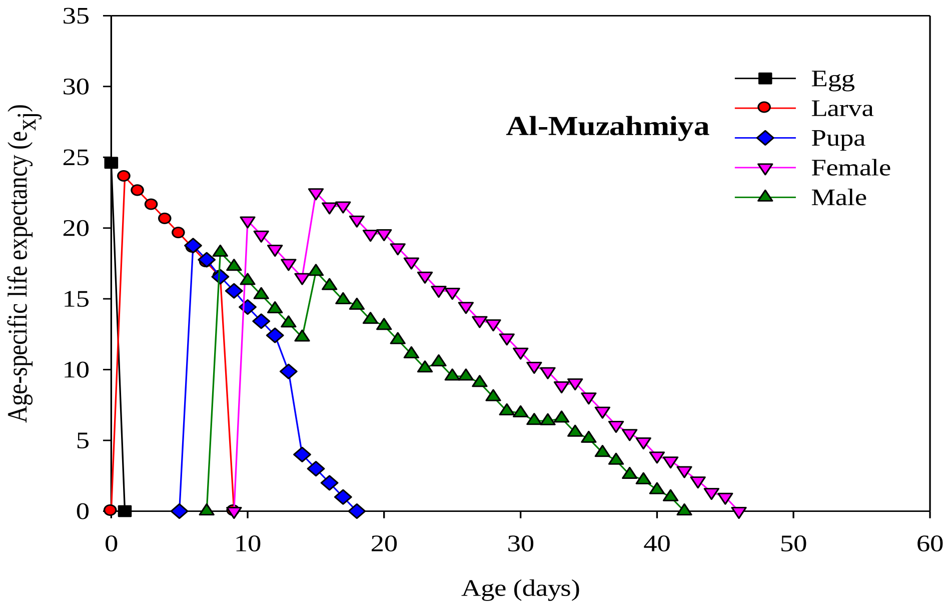

Fig. 4 Age-specific life expectancy (e_{xj}) for the Dirab, Al-Masanie, Al-Washlah, Al-Uraija, and Al-Muzahmiya populations of house fly.

Supplementary files

S1 Life table of *Musca domestica* population collected from Dirab, Saudi Arabia

S2 Life table of *Musca domestica* population collected from Al-Masanie, Saudi Arabia

S3 Life table of *Musca domestica* population collected from Al-Washlah, Saudi Arabia

S4 Life table of *Musca domestica* population collected from Al-Uraija, Saudi Arabia

S5 Life table of Musca domestica population collected from Al-Muzahmiya, Saudi Arabia

Click here to access/download
Supporting Information - Compressed/ZIP File Archive
Life table data.zip

Resistance to insect growth regulators ~~insecticides~~ and age-stage, two-sex life table ~~parameters~~ in ~~house fly,~~ *Musca domestica* from different dairy facilities

Naeem Abbas* and Abdulwahab M. Hafez*

Pesticides and Environmental Toxicology Laboratory, Department of Plant Protection, College of Food and Agriculture Sciences, King Saud University, P.O. Box 2460, Riyadh 11451, Saudi Arabia

*Corresponding authors:

Email: nnoor.c@ksu.edu.sa, ORCID: 0000-0002-2737-7487 (NA)

Email: hafez@ksu.edu.sa, ORCID: 0000-0003-4290-5548 (AH)

Abstract

Among the vectorial insect pests, the domestic house fly (*Musca domestica* L., Diptera: Muscidae) is a ubiquitous livestock pest with the ability to develop resistance and adapt to diverse climates. Successful management of the house fly in various locations requires information about its resistance development and life table ~~traits~~features. The status of insect growth regulators resistance and life table ~~traits~~ features on the basis of age, stage, and two sexes of the house fly from five different geographical locations of Riyadh, Saudi Arabia: Dirab, Al-Masanie, Al-Washlah, Al-Uraija and Al-Muzahmiya were therefore investigated. The range of resistance levels were 3.77–8.03-fold for methoxyfenozide, 5.50–29.75 for pyriproxyfen, 0.59–2.91-fold for cyromazine, 9.33–28.67-fold for diflubenzuron, and 1.63–8.25-fold for triflumuron in five populations of house fly compared with the susceptible strain. Analysis of life history parameters—such as survival rate, larval duration, pupal duration, pre-female duration, pre-male duration, adult and total pre-oviposition periods, longevity of male, oviposition days, female ratio, and fecundity female^{-1}—revealed significant variations among the field populations. Additionally, demographic ~~traits~~features—including the generation time, the finite and intrinsic rates of increase, doubling time, and net reproductive rate—

varied significantly among the field populations. These results will be helpful in planning the management of the house fly in geographically isolated dairies in Saudi Arabia.

Key words: Livestock pest; biological ~~traits~~features; ecology; toxicity; age-specific survivorship; integrated vector management

Introduction

Musca domestica L. (Diptera: Muscidae) is a ubiquitous insect pest of livestock and humans [1, 2] capable of ~~to~~ adapting to a wide range of climates. This insect is commonly known as the house fly and serves as a carrier for many pathogens of public health and veterinary importance [3, 4]. Attempts to control the house fly with a wide range of insecticides have failed because this insect rapidly develops resistance to these chemicals~~Despite the many insecticides developed worldwide in attempts to control the house fly, it has rapidly developed resistance to these chemicals~~ [5-11]. This widespread development of insecticide resistance has necessitated the employment of integrated vector management strategies.

Various control measures are available to manage the house fly, for example, cultural practices, chemicals, and biological agents including fungal/bacterial pathogens and parasitoids/predators or combination of these measures [12, 13]. Insect growth regulators (IGR) comprise an effective chemical tool for integrated pest management due to their properties of high selectivity, killing immature stages, comparative~~ly~~ environmentally safe, and reduced risk to the safety of mammals [14-16]. The juvenile hormone mimic pyriproxyfen inhibits the emergence of adults, cyromazine is a molting disrupter, diflubenzuron and triflumuron are inhibitors of chitin biosynthesis affecting CHS1, and methoxyfenozide is an ecdysone agonist [17]. These insecticides are effectively recommended to manage medical pests, including *M. domestica*, and are strong candidates for integrated pest management due to their different novel mode of actions and less environmental hazards [16, 18-20]. However, resistance to IGRs has been reported previously in house fly [20-25].

Formatted: Font:

The study of life tables involves summarizing age or stage specific birth, death, and reproductive data. Classic models typically focus on females, but we will use both males and females. This methodology is a basic tool in pest management~~The study of life tables on the theory of age, stage, and two sexes of insect pests is a basic and important tool in pest management~~ [26-28]. Comparison of the life tables of geographically separated pest populations aids in the design of effective pest management strategies. Geographic influences such as latitude and longitude and environmental influences such as humidity, photoperiod, and temperature often account for variations in the life tables of insect pests [29, 30]. Geographically and environmentally isolated pest populations have previously been reported to show variations in life tables, for instance, the house fly from urban localities in Pakistan [31]; *Parapoynx crisonalis* (Walker) (Lepidoptera: Pyralidae) [29]; *Plutella xylostella* (Linnaeus) (Lepidoptera: Plutellidae) from China [32]; and *Melanoplus femurrubrum* (De Geer) (Orthoptera: Acrididae) from USA [33]. Variations have also emerged in pesticide resistance in the house fly, *Anopheles stephensi* Liston (Diptera: Culicidae); *Oxycarenus hyalinipennis* Costa (Hemiptera: Lygaidae); *Amrasca devastans* (Distant) (Hemiptera: Cicadellidae); and *Dysdercus koenigii* (Fabricius) (Hemiptera: Pyrrhocoridae) [5, 7, 20, 34-39].

Development of resistance in ~~Management strategies for the development of resistance to insecticides depend primarily on environmental factors and s~~spatial dispers~~al of~~ed pests is important to consider in resistant management strategies. For example, the populations of house fly from Punjab, Pakistan showed significant variations in resistant levels to various insecticides under different environments such as dairy (open) and poultry farms (enclosed) [5, 40]. The knowledge of insecticide resistance and life table variations in the house fly from geographically separated dairies is very important in the design of effective management plans in different regions. Resistance to insect growth regulator insecticides and variations in

the life history ~~traits~~ features of house fly from different originated dairies in Saudi Arabia are currently unexplored. Therefore, the present experiment was conducted to obtain an understanding of the resistance status and life table on the basis of age, stage and two sexes of the house fly collected from different dairies in Saudi Arabia.

Materials and methods

Ethics approval

No specific permit was required to collect house fly samples from the dairy farms, as these farms were privately owned and collections were made merely by personal communication with the owners.

Collections of house fly populations

Approximately 150-200 adult house flies (of mixed sexes) were trapped in plastic jars (33 cm ×19 cm) ~~after attraction by a mixture of dried milk and sugar~~ from five dairy farms located in Riyadh, Saudi Arabia: Dirab (latitude 24.49° N, 46.62° E; elevation 576 m), Al-Masanie (latitude 24.58° N, 46.73° E; elevation 581 m), Al-Uraija (latitude 24.63° N, 46.66° E; elevation 612 m), Al-Washlah (latitude 24.54° N, 46.72° E; elevation 643 m), and ~~Al-Masanie (latitude 24.58° N, 46.73° E; elevation 581 m),~~ Al-Muzahmiya (latitude 24.47° N, 46.27° E; elevation 764 m)~~, and Al-Uraija (latitude 24.63° N, 46.66° E; elevation 612 m)~~. At these dairies, cultural practices (sanitation) and about 8-10 number of insecticide sprays from pyrethroids, organophosphates, and new chemistry classes per season are being used for the control of dairy pests (Personal communication).

Laboratory rearing of house fly populations

The rearing protocol of the house fly was adopted according to Abbas et al. [41] with some modifications. After collection, each population was shifted in a separate transparent cage

(40×40 cm) in the laboratory and reared for ~~three~~ one generation to get uniform insects~~s~~. An adult diet (sugar + powdered milk at a 1:1 ratio, weighed in g) and water-soaked cotton wicks placed in 9 cm plastic petri dishes were provided for adult food. Adults were provided with fresh food ~~after~~ every two days. Cotton wicks were moistened daily ~~with a micro-syringe (5 ml)~~ and replaced at two days interval. A paste of wheat bran, yeast, sugar and milk at a ratio of 20:5:1.5:1.5 (g), respectively, mixed with 120 mL water, was prepared in plastic cups (500 ml) and placed in adult cages for fecundity after two days rearing in the laboratory. Plastic cups with eggs were removed from adult cages daily and covered with a muslin cloth to avoid larval escape. When larvae had consumed the diet in the plastic cups, they were shifted into glass beakers with larval medium, where they became pupae. The emerged adults were shifted to cages to proceed to the next generation. All populations were maintained at 27±2 ºC, 65±5% humidity and a 12:12 h (L:D) photoperiod in the laboratory.

Insecticides

Commercial-grade formulated IGR groups including pyriproxyfen (IRAC # 7C, Admiral 10EC, Sumitomo Chemicals, Japan), diflubenzuron (IRAC # 15, Diflon 250WP, Saudi Delta Company, SA), triflumuron (IRAC # 15, Starycide 480SC, Bayer Crop Sciences, Germany), cyromazine (IRAC # 17, Novasat 75WP, Astranova Chemicals, SA), and methoxyfenozide (IRAC # 18, Runner 24SC, Dow Agro-sciences, UK).

Larval bioassays

The toxicity of IGR insecticides to the larvae of different dairy populations of *M. domestica* was determined through a diet incorporation bioassay following the method of Abbas et al. [20]. Different concentrations of each of the tested IGRs were incorporated into the larval medium (consisting of wheat bran, yeast, milk powder, and sugar at a ratio of 20:5:5:1.5:1.5 g, respectively). Five concentrations (causing mortality from >0% to <100%) of each IGR with

four replicates of each were used in each bioassay. In total, 10 second instar larvae in each replicate, 40 larvae in each concentration, and 200 larvae were used in each bioassay. In the control treatment, 40 larvae with four replicates (10 larvae per replicate) were provided with larval medium without any IGR insecticide concentrations. All bioassays were conducted under the above mentioned constant conditions. Data were recorded at the emergence of adults after 3 weeks of the bioassay. Larvae that failed to transform into adults were considered as dead.

Life table construction

To construct the life table of house fly, 100 freshlynewly laid eggs at different days were randomly collected from different egg batches at one day of each mass population and were placed in plastic cups (500 ml) supplied with artificial larval diet aforementioned above. Single egg was considered as a replicate for each population [42]. The cups were enclosed with muslin cloth to prevent larval escape and arranged into three replicates. Each population was reared separately. Hatched larvae were reared in cups on provided diet until pupation, and their developmental periods were recorded. Freshly emerged adults within 24 h were sexed and one male and one female placed into plastic jars (15 cm x 11 cm)Freshly emerged adults within 24 h were sexed at 1:1 ratio into plastic jars (15 cm × 11 cm). Larval medium was provided daily in 9 cm petri dishes for oviposition. Eggs were counted daily till the death of females and egg hatchability was recorded. The female ratio, adult longevity, and oviposition days were also recorded. All the parameters wereThe experiment was conducted under the aforesaid laboratory conditions.

For each strain, the following parameters were calculated according to Chi and Su [43] and Tuan et al. [27]. Briefly, the age-specific survival rate (‘l_x)’ was determined as follows:

$$l_x = \sum_{j=1}^{m} s_{xj}$$

The age-stage-specific fecundity (m_x) was determined as follows:

$$m_x = \frac{\sum_{j=1}^{m} s_{xj} f_{xj}}{\sum_{j=1}^{m} s_{xj}}$$

The net reproductive rate (Ro) was calculated as follows:

$$R_O = \sum_{x=1}^{\infty} l_x m_x$$

The intrinsic rate of increase (r) was assessed by the Lotka–Euler equation with the age indexing zero as follows:

$$r = \sum_{x=0}^{\infty} e^{-r(x+1)} l_x m_x = 1$$

The finite rate of increase (λ) was determined as:

$$\lambda = e^r$$

The generation time (T) was determined as follows:

$$T = \frac{\ln (Ro)}{r}$$

The life expectancy (e_{xj}) was calculated as follows:

$$e_{xj} = \sum_{i=x}^{n} \sum_{j=y}^{m} s'_{ij}$$

The reproductive value (v_{xj}) was detected as follows:

$$v_{xj} = \frac{e^{-r(x+1)}}{s_{xj}} \sum_{i=x}^{n} e^{-r(i+1)} \sum_{j=y}^{m} s'_{ij} f_{ij}$$

The gross reproduction rate (GRR) was calculated as:

$$GRR = \sum m_x$$

Data analyses

The bioassay data ~~was~~ were analyzed using the POLO Plus software [44] to determine the values of median lethal concentration (LC_{50}). In each bioassay, the mortality rates were corrected by the mortality rate obtained in the control treatment using the formula of Abbott [45], if required. Resistance ratios (RRs) were calculated as: LC_{50} of field population/LC_{50} of susceptible strain. The resistance levels were scaled as mention by Ahmad et al. [46]; RR = 1 (susceptibility), RR = 2–10 (~~tolerance~~ low resistance), RR = 11–30 (moderate resistance), RR = 31–100 (high resistance), and RR > 100 (very high resistance).

The life table data were analyzed with the TWO-SEX-MS Chart program [47] established on the basis of two sex life table principle [28, 48]. The variances and standard errors (SE) of life history ~~traits~~ features were determined by paired bootstrap test with 100,000 replicates at $P \leq 0.05$ using the TWOSEX-MS Chart [47]. The parameters l_x, s_{xj}, f_x, m_x, l_xm_x, v_{xj}, and e_{xj} were graphed with Sigma Plot 11.0.

Results

Resistance of *M. domestica* larvae to IGRs

~~Tolerance to l~~Low resistance against methoxyfenozide (3.77–8.03-fold) and triflumuron (1.63–8.25-fold) was detected in all the five populations of *M. domestica* in comparison to the susceptible strain. Populations collected from the Dirab, Al-Masanie, and Al-Uraija had moderate resistance (10.75–29.75-fold) to pyriproxyfen, while the Al-Washlah and Al-Muzahmiya populations had ~~tolerance to~~ low resistance levels (5.50–8.25-fold), compared with the susceptible strain. Susceptibility to cyromazine was found in all the tested populations (0.59–1.32-fold), except the Al-Masanie population which had ~~tolerance to~~ low resistance (2.91-fold). Populations collected from the Dirab, Al-Washlah, Al-Masanie, and Al-Muzahmiya had moderate resistance (13.67–28.67-fold) to diflubenzuron, while the Al-Uraija population had ~~tolerance to~~ low resistance level (9.33-fold), compared with the susceptible strain (Table 1).

Life history parameters of of house flies from dairiesdairy house flies

The larval durations of all the tested populations were significantly different to from each other ($P < 0.05$). The pupal duration of the Al-Uraija population was significantly longer than that of the Dirab, Al-Masanie, Al-Washlah and Al-Muzahmiya populations ($P < 0.05$). The egg to adult duration for male flies from the Al-Masanie population was significantly longer than for flies from all other areas except DirabThe pre male duration of the Al-Masanie was significantly longer compared to the all other tested populations except Dirab ($P < 0.05$). Similarly, the egg to adult duration for fpre female duration flies of from the Al-Masanie population was significantly longer than for flies from all other areascompared to the all other tested populations ($P < 0.05$). The male total longevity in the Al-Uraija and Al-Muzahmiya populations was significantly shorter than those of the Dirab, Al-Masanie, and Al-Washlah populations ($P < 0.05$). The female total longevity in the Al-Washlah, Al-Uraija, and Al-Muzahmiya populations was significantly shorter ($P < 0.05$) than that of the Al-Masanie population (Table 2).

The TPOP total-pre-oviposition period (TPOP) and APOP adult-pre-oviposition period (APOP) of the Al-Masanie and Al-Washlah populations were significantly longer than those of the Dirab, Al-Uraija, and Al-Muzahmiya populations ($P < 0.05$). The oviposition days were significantly reduced in the Al-Washlah population compared with that of the Al-Muzahmiya population ($P < 0.05$), but similar with the other tested populations. The female ratio in the Al-Uraija population was significantly lower compared with the Dirab and Al-Washlah populations ($P < 0.05$). Whereas the reproductive female ratio was significantly lower in the Al-Washlah population compared with the other populations ($P < 0.05$). The fecundity/female was significantly lower in the Al-Washlah population than that of the Al-Masanie and Al-Muzahmiya populations ($P < 0.05$), but did not differ from that of the Dirab and Al-Uraija populations (Table 2).

Demographic life table ~~traits~~ features of house flies from dairies~~of dairy house flies~~

The intrinsic rates of increase (r) in the Al-Masanie (0.013) and Al-Washlah (0.12) populations were significantly lower ($P < 0.05$) than that of the Dirab (0.17), Al-Uraija (0.18), and Al-Muzahmiya (0.19) populations. The finite rates of increase (λ) of the Al-Masanie and Al-Washlah (1.13) populations were significantly lower than that of the Dirab (1.18), Al-Uraija (1.19), and Al-Muzahmiya (1.21) populations ($P < 0.05$). The net reproductive rate (R_0) in the Al-Muzahmiya population (89.32) was significantly higher than that of the Al-Masanie (39.52) and Al-Washlah (33.04) populations ($P < 0.05$), and similar to that of the Dirab (74.50) and Al-Uraija (64.35) populations. The generation time (T) in the Al-Masanie (29.43) and Al-Washlah (29.80) populations was significantly longer than that of the Dirab (26.05), Al-Uraija (23.54), and Al-Muzahmiya (24.08) populations ($P < 0.05$). The doubling time (DT) was shorter in the Al-Uraija (3.92) and Al-Muzahmiya (3.72) populations ($P < 0.05$) than that of the Al-Washlah population (5.91), but similar to that of the Dirab (4.19) and Al-Masanie (5.55) populations. Among all the tested populations, the GRR (gross reproduction rate) was similar ($P > 0.05$, Table 3).

Age-stage-specific survival rates (s_{xj}) of house flies from dairies~~of dairy house flies~~

The parameter s_{xj} refers to the possible survival of newly born eggs to age x and development to stage j (Fig. 1). In all the populations the peak value of s_{xj} for larvae was similar. However, the highest peak values of s_{xj} for pupae in the Dirab (0.80) and Al-Washlah (0.70) populations were lower than those of the Al-Masanie (0.89), Ai-Uraija (0.99), and Al-Muzahmiya (0.97) populations. Similarly, the highest peak values of s_{xj} for females and males in the Dirab (0.25 and 0.24, respectively), Al-Uraija (0.21 and 0.49, respectively), and Al-Muzahmiya (0.29 and 0.36, respectively) populations were higher than those for the Al-Masanie (0.11 and 0.14, respectively) and Al-Washlah (0.19 and 0.13, respectively) populations.

Age-specific survival rates (l_x), fecundities (m_x, f_x), and maturity (l_xm_x) of house flies from dairies~~of dairy house flies~~

The parameters l_x, f_x, m_x and l_xm_x were determined for all populations (Fig. 2). Among all populations, non-significant differences were found in the highest peak values for l_x. The highest peaks of f_x in the Dirab (40.67 eggs female^{-1}day^{-1}) and Al-Muzahmiya (40.06 eggs female^{-1}day^{-1}) populations were lower than those in the Al-Masanie (44.33 eggs female^{-1}day^{-1}), Al-Washlah (43.75 eggs female^{-1}day^{-1}), and Al-Uraija (55.67 eggs female^{-1}day^{-1}) populations, while the highest peaks of m_x in the Dirab (19 eggs individual^{-1}day^{-1}), Al-Masanie (20.71 eggs individual^{-1}day^{-1}), and Al-Uraija (21.97 eggs individual^{-1}day^{-1}) populations were lower than those in the Al-Washlah (25 eggs individual^{-1}day^{-1}) and Al-Muzahmiya (34.43 eggs individual^{-1}day^{-1}) populations. Additionally, the peak times of l_x, f_x and m_x for the Dirab (45th day), Al-Masanie (29th day), Al-Washlah (43th day), Al-Uraija (20th day), and Al-Muzahmiya (31th day) populations varied significantly among populations. Similarly, the highest peak values for l_xm_x in the Al-Masanie (4.35 at 24th day) and Al-Washlah (2.68 at 25th day) populations were lower than those in the Dirab (6.08 at 21th day), Al-Uraija (8.35 at 20th day), and Al-Muzahmiya (7.12 at 21th day) populations.

Age-stage-specific reproductive values (v_{xj}) of house flies from dairies~~of dairy house flies~~

The v_{xj} reveals the reproductive value of insects at the age x and stage j in terms of future progeny (Fig. 3). The peak v_{xj} values for larvae were 6.54, 3.91, 4.11, 3.44, and 5.52 day^{-1} in the Dirab, Al-Masanie, Al-Washlah, Al-Uraija, and Al-Muzahmiya populations, respectively. The peak v_{xj} values for pupae in the Dirab (12.35 day^{-1}) Al-Masanie (7.49 day^{-1}), Al-Uraija (11.41 day^{-1}), and Al-Muzahmiya (11.56 day^{-1}) populations were lower than those in the Al-Washlah population (18.00 day^{-1}). The peak v_{xj} values for females in the Al-Masanie (166.7 day^{-1}), Al-Washlah (160.92 day^{-1}) and Al-Uraija (169.90 day^{-1}) populations were significantly higher than those in the Dirab (126.12 day^{-1}) and Al-Muzahmiya (132.47 day^{-1}) populations.

The duration was shorter in the Al-Uraija and Al-Muzahmiya populations than that in the Dirab, Al-Masanie, and Al-Washlah populations.

Age-stage-specific life expectancy (e_{xj}) of ~~dairy~~ house flies from dairies

The e_{xj} refers to the expected days over which an insect at age x and stage j will survive after age x shown in Fig. 4. The e_{xj} values for eggs were 22.13, 21.33, 16.48, 21.10, and 24.61 days in the Dirab, Al-Masanie, Al-Washlah, Al-Uraija, and Al-Muzahmiya populations, respectively. In the Al-Uraija and Al-Washlah populations, the peak e_{xj} values for larvae were 20.1 and 20.69 days, respectively, which were shorter than those in the Dirab (25.16 days), Al-Masanie (21.72), and Al-Muzahmiya (23.61 days) populations. The peak e_{xj} values for pupae were 19.16, 14.72, 17.69, 16.1, and 18.76 days in the Dirab, Al-Masanie, Al-Washlah, Al-Uraija, and Al-Muzahmiya populations, and each of these values differed significantly. The peak e_{xj} values for the female and male house fly differed significantly in the tested populations—Al-Masanie (26.1 days and 24.73 days, respectively), Al-Washlah (24.88 days and 29.46 days, respectively), Al-Muzahmiya (21.60 days and 16.13 days, respectively), Dirab (19.80 days and 19.81 days, respectively), and Al-Uraija (18.80 days and 18.30 days, respectively).

Discussion

In the current study, the five populations of house fly collected from dairy facilities showed susceptibility to cyromazine, ~~tolerance to~~ low resistance to methoxyfenozide and triflumuron, ~~tolerance to~~ low–moderate resistance to pyriproxyfen and diflubenzuron in comparison to the susceptible strain. The insect populations that showed more than tenfold resistance to insecticides are known to be resistant [5, 49]. In this study, less than tenfold resistance to methoxyfenozide, cyromazine and triflumuron in all tested populations, pyriproxyfen in two populations, and diflubenzuron in one population were detected. These populations were considered tolerant rather than resistant. Previously, resistance to these insecticides has been

~~occurred~~ found in house fly [20-22], *Phenacoccus solenopsis* Tinsley (Homoptera: Pseudococcidae) [50], *Spodoptera litura* (Fabricius) (Lepidoptera: Noctuidae) [51], and *Spodoptera frugiperda* (J.E. Smith) (Lepidoptera: Noctuidae) [52]. However, this is the first susceptibility report of house fly populations to IGRs from dairy facilities in Riyadh, Saudi Arabia. Among the five tested IGRs, methoxyfenozide, cyromazine, and triflumuron were found to be the most toxic larvicides against house fly larvae. Therefore, these larvicides could be the most promising agents for the integrated vector management system in Saudi Arabia. However, the potential cross-resistance from other chemical classes [17] should be considered when aiming to design a successful IGRs rotation pattern which requires further studies to prolong the effectiveness of these larvicides. Additionally, use of cultural practices (sanitation) and biological control agents with inclusion of insecticides for the control of house fly should be considered as an integrated pest management tool [12, 13].

The adaptation of an insect pest to an altered environment and insecticide applications depends on its life table parameters, and evidence defining its population dynamics is crucial in formulating an effective pest management plan. The present study was conducted on the five house fly populations from different dairy farms in Riyadh, Saudi Arabia, to explore their life table dynamics. Results revealed prominent variations in the larval duration, pupal duration, pre-adult male and female duration, oviposition days, longevity of males and females, APOP, TPOP, female ratio, and fecundity female^{-1}. The reason for the differences in life history ~~traits~~ features could be attributed to altered elevations, latitudes, and environmental factors—for instance, temperature and humidity—favoring the adaptation of the house flies. Similar to those in the current results, significant variations in life history parameters have been reported in several differing isolated pest populations [29-31, 53, 54]. For example, the developmental time of *P. xylostella* populations from higher latitudes was longer than that from lower latitudes [32]. In contrast, Shirai [54] reported no difference in the

immature developmental time among nine geographically separate *P. xylostella* populations. The larval duration in *Colaphellus bowringi* Baly (Coleoptera: Chrysomelidae) was increased with increasing latitudes [30], but decreased in geometrid moths; *Cabera exanthemata* (Scopoli), *Lomaspilis marginata* (Linnaeus), *Chiasmia clathrata* (Linnaeus), and *Cabera pusaria* (Linnaeus) (Lepidoptera: Geometridae) [53]. Chen et al. [29] reported a higher net reproductive rate at 24 ˚C than at other tested temperatures in *P. crisonalis*. In house fly populations from Pakistan, the immature developmental time was shorter and pupal weights were heavier in those from lower latitudes with hot climates [31]. The aforementioned variations among different insect pests could be due to the varied latitudes and favorable environments responsible for the flexibility of insect pests in their particular regions [30, 55], and/or the fitness costs accompanying with the development of insecticide resistance [1, 2, 56] due to pesticide application for the control of the house fly by dairy farmers.

The present results exhibited variations in the demographic parameters (r, λ, R_O, T, and DT) among the five house fly populations. The r and λ of the Al-Masanie and Al-Washlah populations were significantly lower than those in other tested field populations. The Al-Muzahmiya population exhibited the highest *Ro* among all of the field populations. The mean generation time of the Al-Uraija and Al-Muzahmiya populations was significantly shorter than that of other tested populations, while the doubling time of the Al-Washlah population was higher than those of the Al-Uraija and Al-Muzahmiya populations. The parameters r, λ, and R_O provide the growth potential estimation of pests—a wider insight than that provided by individual life history ~~traits~~ features [2]. Because the parameters r and λ depend upon the fecundity and growth of individuals, therefore differences in these parameters might affect the expansion rates of populations [2, 57, 58]. The lower rates of increase in the populations in this study could be attributable to lower fecundity female^{-1}. ~~Furthermore, the differences in latitudes, longitudes and elevations of the dairy farms might be responsible for the variations~~

in the demographic parameters of the various house fly populations. In agreement with present results, significant variations in demographic life table parameters of pests have previously been attributed to the aforesaid factors [27],reported in *P. crisonalis* [29], *M. femurrubrum* [33], and *P. xylostella* [32],

The parameters s_{xj}, l_x, f_x, m_x, l_xm_x, $v_{xj,}$ and e_{xj} are important indicators for evaluating the biological fitness of insect pest populations. Similar insect pests under altered environments have different survival rates, reproduction abilities, and life spans, all of which reflect specific environmental effects and species-specific parameters [59, 60]. The present results for s_{xj}, l_x, f_x, m_x, l_xm_x, $v_{xj,}$ and e_{xj} revealed significant variations in house fly populations from five different dairy farms. Because of differing environmental factors—for instance, temperature—and extents of insecticide exposure, significant variations in these parameters have previously been documented in a range of pests, including *P. crisonalis* [29], *Sogatella furcifera* (Horvath) (Hemiptera: Delphacidae) [60], *Bradysia odoriphaga* Yang and Zhang (Diptera: Sciaridae) [42], *Phthorimaea operculella* Zeller (Lepidoptera: Gelechiidae) [61], *S. litura* [27], and *Tetranychus urticae* Koch (Acari: Tetranychidae) [62].

Effectiveness and implementation of control measures against insect pests depend on knowledge of the life table attributes of any pest in its respective environment [31, 32]. Our results provide useful insights about the age, stage, and two sexes based life table variation in house fly populations from Riyadh, Saudi Arabia. The higher *r* and λ values and shorter T values in the Dirab, Al-Uraija, and Al-Muzahmiya populations revealed similarities among these populations, while they differed significantly from the Al-Masanie and Al-Washlah populations. In the house fly, a shorter life span predicts the development of insecticide resistance; therefore present data might be helpful in predicting the growth rates of house fly populations in geographically separated dairy farms and in formulating timely management strategies for this pest in specific dairies.

Acknowledgments

The authors extend their appreciation to the Deputyship for Research & Innovation, "Ministry of Education" in Saudi Arabia for funding this research work through the project number IFKSURG-1441-480.~~The authors extend the appreciation for the Deanship of Scientific Research at King Saud University for funding this work through the research group No. RGP-1441-480.~~ The authors thank the Deanship of Scientific Research and Researchers Support and Services Unit (RSSU) at King Saud University for their technical support. Authors would like also to thank the researchers and technicians from Pesticides and Environmental Toxicology Laboratory: Ahmed Mohammed Dabo and Safwat Gamal Sabra for their help in collecting and maintaining the house fly field populations in the laboratory and for other laboratory work.

Conflict of interest

The authors have declared no conflict of interest.

References

1. Abbas N, Shah RM, Shad SA, Azher F. Dominant fitness costs of resistance to fipronil in *Musca domestica* Linnaeus (Diptera: Muscidae). Vet Parasitol. 2016;226:78-82.
2. Shah RM, Shad SA, Abbas N. Methoxyfenozide resistance of the housefly, *Musca domestica* L. (Diptera: Muscidae): cross-resistance patterns, stability and associated fitness costs. Pest Manag Sci. 2017;73(1):254-61.
3. Fasanella A, Scasciamacchia S, Garofolo G, Giangaspero A, Tarsitano E, Adone R. Evaluation of the house fly *Musca domestica* as a mechanical vector for an anthrax. Plos One. 2010;5(8):e12219.
4. Förster M, Klimpel S, Mehlhorn H, Sievert K, Messler S, Pfeffer K. Pilot study on synanthropic flies (e.g. *Musca, Sarcophaga, Calliphora, Fannia, Lucilia, Stomoxys*) as vectors of pathogenic microorganisms. Parasitol Res. 2007;101(1):243-6.
5. Abbas N, Shad SA, Ismail M. Resistance to conventional and new insecticides in house flies (Diptera: Muscidae) from poultry facilities in Punjab, Pakistan. J Econ Entomol. 2015;108(2):826–33.
6. Abbas N, Ijaz M, Shad SA, Binyameen M. Assessment of resistance risk to fipronil and cross resistance to other insecticides in the *Musca domestica* L. (Diptera: Muscidae). Vet Parasitol. 2016;223:71-6.

7. Khan HAA, Akram W, Fatima A. Resistance to pyrethroid insecticides in house flies, *Musca domestica* L., (Diptera: Muscidae) collected from urban areas in Punjab, Pakistan. Parasitol Res. 2017;116(12):3381-5.
8. Freeman JC, Ross DH, Scott JG. Insecticide resistance monitoring of house fly populations from the United States. Pestic Biochem Physiol. 2019;158:61-8.
9. Li Q, Huang J, Yuan J. Status and preliminary mechanism of resistance to insecticides in a field strain of house fly (*Musca domestica*, L). Rev Bras Entomol. 2018;62(4):311-4.
10. Shah RM, Shad SA. House fly resistance to chlorantraniliprole: cross resistance patterns, stability and associated fitness costs. Pest Manag Sci. 2020;76(5):1866-73.
11. Abbas N, Khan HAA, Shad SA. Resistance of the house fly *Musca domestica* (Diptera: Muscidae) to lambda-cyhalothrin: mode of inheritance, realized heritability, and cross-resistance to other insecticides. Ecotoxicology. 2014;23(5):791-801.
12. Malik A, Singh N, Satya S. House fly (*Musca domestica*): a review of control strategies for a challenging pest. Journal of Environmental Science and Health Part B. 2007;42(4):453-69.
13. Farooq M, Freed S. Infectivity of housefly, *Musca domestica* (Diptera: Muscidae) to different entomopathogenic fungi. Braz J Microbiol. 2016;47(4):807-16.
14. Tunaz H, Uygun N. Insect growth regulators for insect pest control. Turkish Journal of Agriculture and Forestry. 2004;28(6):377-87.
15. Mian LS, Mulla MS. Biological and environmental dynamics of insect growth regulators (IGRs) as used against Diptera of public health importance. Residue Reviews: Springer; 1982. p. 27-112.
16. Kočišová A, Petrovský M, Toporčák J, Novák P. The potential of some insect growth regulators in housefly (*Musca domestica*) control. Biologia, Bratislava. 2004;59(5):661-8.
17. IRAC. IRAC: Mode of action classification scheme, Version 9.4, .www.irac-online.org › documents › moa-classification. 2020:pp, 1-26.
18. Shah RM, Abbas N, Shad SA, Sial AA. Selection, resistance risk assessment, and reversion toward susceptibility of pyriproxyfen in *Musca domestica* L. Parasitol Res. 2015;114(2):487-94.
19. Matsumura F. Studies on the action mechanism of benzoylurea insecticides to inhibit the process of chitin synthesis in insects: A review on the status of research activities in the past, the present and the future prospects. Pestic Biochem Physiol. 2010;97(2):133-9.
20. Abbas N, Shad SA, Shah RM. Resistance status of *Musca domestica* L. populations to neonicotinoids and insect growth regulators in Pakistan poultry facilities. Pak J Zool. 2015;47(6):1663-71.
21. Khan HAA, Akram W, Arshad M, Hafeez F. Toxicity and resistance of field collected *Musca domestica* (Diptera: Muscidae) against insect growth regulator insecticides. Parasitol Res. 2016;115(4):1385-90.
22. Bell HA, Robinson KA, Weaver RJ. First report of cyromazine resistance in a population of UK house fly (*Musca domestica*) associated with intensive livestock production. Pest Manag Sci. 2010;66(7):693-5.
23. Tang JD, Caprio MA, Sheppard DC, Gaydon DM. Genetics and fitness costs of cyromazine resistance in the house fly (Diptera: Muscidae). J Econ Entomol. 2002;95(6):1251-60.
24. Shah RM, Shad SA, Abbas N. Mechanism, stability and fitness cost of resistance to pyriproxyfen in the house fly, *Musca domestica* L. (Diptera: Muscidae). Pestic Biochem Physiol. 2015;119:67-73.
25. Shah RM, Abbas N, Shad SA, Binyameen M. Determination of the genetic and synergistic suppression of a methoxyfenozide-resistant strain of the house fly *Musca domestica* L. (Diptera: Muscidae). Neotrop Entomol. 2018;47(5):709-15.

26. Chi H, You M, Atlıhan R, Smith CL, Kavousi A, Özgökçe MS, et al. Age-stage, two-sex life table: an introduction to theory, data analysis, and application. Entomol Gen. 2020;40(2):102-23.
27. Tuan SJ, Lee CC, Chi H. Population and damage projection of *Spodoptera litura* (F.) on peanuts (*Arachis hypogaea* L.) under different conditions using the age-stage, two-sex life table. Pest Manag Sci. 2014;70(5):805-13.
28. Chi H. Life-table analysis incorporating both sexes and variable development rates among individuals. Environ Entomol. 1988;17(1):26-34.
29. Chen Q, Li N, Wang X, Ma L, Huang J-B, Huang G-H. Age-stage, two-sex life table of *Parapoynx crisonalis* (Lepidoptera: Pyralidae) at different temperatures. PloS one. 2017;12(3):e0173380.
30. Tang J, He H, Chen C, Fu S, Xue F. Latitudinal cogradient variation of development time and growth rate and a negative latitudinal body weight cline in a widely distributed cabbage beetle. PloS one. 2017;12(7):e0181030.
31. Khan HAA, Khan MU, Nasiba A, Riaz S, Altaf M. Geographical variations in life histories of house flies, *Musca domestica* (Diptera: Muscidae), in Punjab, Pakistan. J Med Entomol. 2019;56(5):1225-30.
32. Pan Q-J, Chen L, Lin X-L, Ridsdill-Smith TJ, Liu T-X. Geographical variations in life histories of *Plutella xylostella* in China. J Pest Sci. 2014;87(4):659-70.
33. Parsons SMA, Joern A. Life history traits associated with body size covary along a latitudinal gradient in a generalist grasshopper. Oecologia. 2014;174(2):379-91.
34. Saeed R, Abbas N, Razaq M, Mahmood Z, Naveed M, Rehman HMU. Field evolved resistance to pyrethroids, neonicotinoids and biopesticides in *Dysdercus koenigii* (Hemiptera: Pyrrhocoridae) from Punjab, Pakistan. Chemosphere. 2018;213:149-55. doi: 10.1016/j.chemosphere.2018.09.042. PubMed PMID: WOS:000448493700016.
35. Saeed R, Razaq M, Abbas N, Jan MT, Naveed M. Toxicity and resistance of the cotton leaf hopper, *Amrasca devastans* (Distant) to neonicotinoid insecticides in Punjab, Pakistan. Crop Prot. 2017;93:143-7.
36. Ullah S, Shad SA, Abbas N. Resistance of dusky Cotton bug, *Oxycarenus hyalinipennis* Costa (Lygaidae: Hemiptera), to conventional and novel chemistry insecticides. J Econ Entomol. 2016;109(1):345-51.
37. Khan HAA, Akram W, Lee S. Resistance to selected pyrethroid insecticides in the malaria mosquito, *Anopheles stephensi* (Diptera: Culicidae), from Punjab, Pakistan. J Med Entomol. 2018;55(3):735-8.
38. Kaufman PE, Nunez SC, Mann RS, Geden CJ, Scharf ME. Nicotinoid and pyrethroid insecticide resistance in houseflies (Diptera: Muscidae) collected from Florida dairies. Pest Manag Sci. 2010;66(3):290-4.
39. Wang J-N, Hou J, Wu Y-Y, Guo S, Liu Q-M, Li T-Q, et al. Resistance of house fly, *Musca domestica* L. (Diptera: Muscidae), to five insecticides in Zhejiang Province, China: The situation in 2017. Canadian Journal of Infectious Diseases and Medical Microbiology. 2019;2019:1-10.
40. Khan HAA, Akram W, Shad SA. Resistance to conventional insecticides in Pakistani populations of *Musca domestica* L. (Diptera: Muscidae): a potential ectoparasite of dairy animals. Ecotoxicology. 2013;22:522-7.
41. Abbas N, Khan HAA, Shad SA. Cross-resistance, genetics, and realized heritability of resistance to fipronil in the house fly, *Musca domestica* (Diptera: Muscidae): a potential vector for disease transmission. Parasitol Res. 2014;113(4):1343–52.
42. Gul H, Ullah F, Biondi A, Desneux N, Qian D, Gao X, et al. Resistance against clothianidin and associated fitness costs in the chive maggot, *Bradysia odoriphaga*. Entomol Gen. 2019;39(2):81-92.

43. Chi H, Su H-Y. Age-stage, two-sex life tables of *Aphidius gifuensis* (Ashmead) (Hymenoptera: Braconidae) and its host *Myzus persicae* (Sulzer) (Homoptera: Aphididae) with mathematical proof of the relationship between female fecundity and the net reproductive rate. Environ Entomol. 2006;35(1):10-21.
44. LeOra S. Poloplus, a user's guide to probit or logit analysis. LeOra Software, Berkeley, CA. 2003.
45. Abbott W. A method of computing the effectiveness of an insecticide. J Econ Entomol. 1925;18(2):265-7.
46. Ahmad M, Iqbal Arif M, Ahmad M. Occurrence of insecticide resistance in field populations of *Spodoptera litura* (Lepidoptera: Noctuidae) in Pakistan. Crop Prot. 2007;26(6):809-17.
47. Chi H. TWOSEX-MSChart: a computer program for the age-stage, two-sex life table analysis. http://140120197173/Ecology/prod02htm. 2019.
48. Chi H, Yang T-C. Two-sex life table and predation rate of *Propylaea japonica* Thunberg (Coleoptera: Coccinellidae) fed on *Myzus persicae* (Sulzer) (Homoptera: Aphididae). Environ Entomol. 2003;32(2):327-33.
49. Valles SM, Koehler PG, Brenner RJ. Antagonism of fipronil toxicity by piperonyl butoxide and S, S, S-tributyl phosphorotrithioate in the German cockroach (Dictyoptera: Blattellidae). J Econ Entomol. 1997;90(5):1254-8.
50. Saddiq B, Shad SA, Aslam M, Ijaz M, Abbas N. Monitoring resistance of *Phenacoccus solenopsis* Tinsley (Homoptera: Pseudococcidae) to new chemical insecticides in Punjab, Pakistan. Crop Prot. 2015;74:24-9.
51. Ahmad M, Gull S. Susceptibility of armyworm *Spodoptera litura* (Lepidoptera: Noctuidae) to novel insecticides in Pakistan. The Canadian Entomologist. 2017;149(5):649-61.
52. Gutiérrez-Moreno R, Mota-Sanchez D, Blanco CA, Whalon ME, Terán-Santofimio H, Rodriguez-Maciel JC, et al. Field-evolved resistance of the fall armyworm (Lepidoptera: Noctuidae) to synthetic insecticides in Puerto Rico and Mexico. J Econ Entomol. 2019;112(2):792-802.
53. Kivelä SM, Välimäki P, Carrasco D, Mäenpää MI, Oksanen J. Latitudinal insect body size clines revisited: a critical evaluation of the saw-tooth model. J Anim Ecol. 2011;80(6):1184-95.
54. Shirai Y. Temperature tolerance of the diamondback moth, *Plutella xylostella* (Lepidoptera: Yponomeutidae) in tropical and temperate regions of Asia. Bull Entomol Res. 2000;90(4):357-64.
55. Honek A. Geographical variation in thermal requirements for insect development. Eur J Entomol. 2013;93(3):303-12.
56. Abbas N, Shah RM, Shad SA, Iqbal N, Razaq M. Biological trait analysis and stability of lambda-cyhalothrin resistance in the house fly, *Musca domestica* L. (Diptera: Muscidae). Parasitol Res. 2016;115(5):2073-80.
57. Abbas N, Khan H, Shad SA. Cross-resistance, stability, and fitness cost of resistance to imidacloprid in *Musca domestica* L., (Diptera: Muscidae). Parasitol Res. 2015;114(1):247-55. Epub 2014/10/25. PubMed PMID: 25342464.
58. Abbas N, Shad SA, Razaq M. Fitness cost, cross resistance and realized heritability of resistance to imidacloprid in *Spodoptera litura* (Lepidoptera: Noctuidae). Pestic Biochem Physiol. 2012;103(3):181-8.
59. Zhang P, Zhao Y-H, Wang Q-H, Mu W, Liu F. Lethal and sublethal effects of the chitin synthesis inhibitor chlorfluazuron on *Bradysia odoriphaga* Yang and Zhang (Diptera: Sciaridae). Pestic Biochem Physiol. 2017;136:80-8.

60. Xiang X, Liu S, Wang X, Zhang Y, Gong C, Chen L, et al. Sublethal effects of sulfoxaflor on population projection and development of the white-backed planthopper, *Sogatella furcifera* (Hemiptera: Delphacidae). Crop Prot. 2019;120:97-102.
61. Yuan H-g, Wu S-y, Lei Z-r, Rondon SI, Gao Y-l. Sub-lethal effects of *Beauveria bassiana* (Balsamo) on field populations of the potato tuberworm *Phthorimaea operculella* Zeller in China. Journal of Integrative Agriculture. 2018;17(4):911-8.
62. Wang L, Zhang Y, Xie W, Wu Q, Wang S. Sublethal effects of spinetoram on the two-spotted spider mite, *Tetranychus urticae* (Acari: Tetranychidae). Pestic Biochem Physiol. 2016;132:102-7.

Table 1 Resistance to insect growth regulators in the larvae of different dairy farms *M. domestica* populations

Insecticide	Population	Year	[1]N	Slope ± SE	χ^2 (df)	P	[2]LC_{50}	[3]FL (95%)	[4]RR
Pyriproxyfen	Susceptible (G_{14})	2019	240	1.52 ± 0.19	6.17 (3)	0.10	0.04	0.03–0.06	1.00
	Dirab (G_1)	2019	240	1.63 ± 0.27	5.07 (3)	0.17	1.19	0.26–2.38	29.75
	Al-Masanie (G_1)	2019	240	2.21 ± 0.34	5.98 (3)	0.11	0.70	0.16–1.19	17.50
	Al-Uraija (G_1)	2019	240	1.68 ± 0.32	1.52 (3)	0.68	0.43	0.21–0.63	10.75
	Al-Washlah (G_1)	2019	240	0.80 ± 0.24	1.00 (3)	0.80	0.22	0.01–0.53	5.50
	Al-Muzahmiya (G_1)	2019	240	1.31 ± 0.29	0.77 (3)	0.86	0.33	0.10–0.56	8.25
Diflubenzuron	Susceptible (G_{14})	2019	240	1.49 ± 0.34	2.27 (3)	0.52	0.03	0.01–0.05	1.00
	Dirab (G_1)	2019	240	1.87 ± 0.28	3.75 (3)	0.29	0.41	0.19–0.70	13.67
	Al-Masanie (G_1)	2019	240	1.68 ± 0.24	3.17 (3)	0.37	0.46	0.28–0.74	15.33
	Al-Uraija (G_1)	2019	240	1.04 ± 0.24	0.10 (3)	0.99	0.28	0.10–0.46	9.33
	Al-Washlah (G_1)	2019	240	1.31 ± 0.17	4.45 (3)	0.22	0.86	0.55–1.40	28.67
	Al-Muzahmiya (G_1)	2019	240	1.53 ± 0.24	5.06 (3)	0.17	0.86	0.37–1.78	28.67
Triflumuron	Susceptible(G_{14})	2019	240	0.99 ± 0.32	0.13 (3)	0.99	0.08	0.00–0.26	1.00
	Dirab (G_1)	2019	240	2.24 ± 0.66	0.24 (3)	0.97	0.23	0.04–0.39	2.88
	Al-Masanie (G_1)	2019	240	1.08 ± 0.25	6.05 (3)	0.11	0.62	0.27–3.53	7.75
	Al-Uraija (G_1)	2019	240	1.83 ± 0.30	0.13 (3)	0.99	0.66	0.42–0.89	8.25
	Al-Washlah (G_1)	2019	240	1.67 ± 0.38	0.84 (3)	0.84	0.27	0.08–0.41	3.38
	Al-Muzahmiya (G_1)	2019	240	1.54 ± 0.47	1.03 (3)	0.79	0.13	0.01–0.30	1.63
Cyromazine	Susceptible (G_{14})	2019	240	2.54 ± 0.31	4.76 (3)	0.19	0.22	0.18–0.28	1.00
	Dirab (G_1)	2019	240	2.15 ± 0.27	1.57 (3)	0.67	0.16	0.13–0.21	0.73
	Al-Masanie (G_1)	2019	240	1.82 ± 0.36	4.76 (3)	0.19	0.64	0.43–1.34	2.91
	Al-Uraija (G_1)	2019	240	2.76 ± 0.39	1.30 (3)	0.73	0.29	0.24–0.37	1.32
	Al-Washlah (G_1)	2019	240	2.47 ± 0.32	6.88 (3)	0.08	0.22	0.13–0.40	1.00
	Al-Muzahmiya (G_1)	2019	240	3.43 ± 0.39	6.09 (3)	0.12	0.13	0.11–0.15	0.59
Methoxyfenozide	Susceptible (G_{14})	2019	240	2.13 ± 0.26	5.38 (3)	0.15	4.99	3.91–6.40	1.00
	Dirab (G_1)	2019	240	1.58 ± 0.36	3.26 (3)	0.35	34.55	23.27–80.48	6.92
	Al-Masanie (G_1)	2019	240	3.09 ± 0.45	1.34 (3)	0.72	40.09	33.46–50.03	8.03
	Al-Uraija (G_1)	2019	240	3.25 ± 0.38	0.94 (3)	0.82	21.55	18.25–25.58	4.32
	Al-Washlah (G_1)	2019	240	2.15 ± 0.27	1.57 (3)	0.67	20.79	16.75–26.30	4.17
	Al-Muzahmiya (G_1)	2019	240	1.56 ± 0.24	0.59 (3)	0.90	18.79	14.16–25.48	3.77

[1] Number of tested larvae. [2] Median lethal concentrations in μg/ml. [3] Fiducial limits [4] Resistance ratios.

Table 2 Development duration and life history parameters of five house fly populations collected from dairies in Riyadh, Saudi Arabia

Parameters	N	Dirab ±SEM*	N	Al-Masanie ±SEM*	N	Al-Washlah ±SEM*	N	Al-Uraija ±SEM*	N	Al-Muzahmiya ±SEM*
larvae (d)	80	7.89 ± 0.05 b	89	8.09 ± 0.03 a	70	6.80 ± 0.11 d	100	5.90 ± 0.04 e	100	7.34 ± 0.11 c
pupae (d)	50	5.68 ± 0.09 c	27	6.00 ± 0.11 b	33	5.58 ± 0.12 c	70	6.80 ± 0.06 a	65	5.11 ± 0.14 d
pre-adult ♂ (d)	25	14.60 ± 0.14 ab	16	15.00 ± 0.18 a	13	14.00 ± 0.39 bc	49	13.69 ± 0.11 c	36	12.89 ± 0.28 d
pre-adult ♀ (d)	25	14.56 ± 0.13 b	11	15.00 ± 0.00 a	20	14.35 ± 0.21 b	21	13.57 ± 0.27 c	29	13.35 ± 0.19 c
longevity ♂ (d)	25	32.84 ± 2.37 b	16	33.88 ± 3.56 ab	13	40.46 ± 2.49 a	49	22.57 ± 1.31 c	36	26.28 ± 1.66 c
longevity ♀ (d)	25	32.80 ± 2.19 ab	11	39.73 ± 3.20 a	20	27.00 ± 3.49 b	21	27.81 ± 2.01 b	29	30.52 ± 2.39 b
APOP[a] (d)	19	5.47 ± 0.41 b	10	8.70 ± 0.79 a	~~8~~9	8.75 ± 0.93 a	15	4.80 ± 0.61 b	20	4.45 ± 0.42 b
TPOP[b] (d)	19	19.89 ± 0.42 b	10	23.70 ± 0.79 a	9~~8~~	22.75 ± 0.58 a	15	18.20 ± 0.76 c	20	17.50 ± 0.45 c
oviposition (d)	25	6.42 ± 1.12 ab	11	6.90 ± 1.30 ab	20	5.88 ± 1.03 b	21	7.07 ± 0.70 ab	29	8.95 ± 0.93 a
female ratio	100	0.50 ± 0.007 a	100	0.41 ± 0.01 ab	100	0.61 ± 0.009 a	100	0.30 ± 0.005 b	100	0.45 ± 0.006 ab
rep. female ratio	100	0.76 ± 0.009 a	100	0.91 ± 0.009 a	100	0.40 ± 0.11 b	100	0.71 ± 0.10 a	100	0.69 ± 0.009 a
fecundity/female	25	298.00 ± 69.70 ab	11	359.27 ± 77.79 a	20	165.2 ± 52.32 b	21	306.43 ± 59.48 ab	29	308.00 ± 52.70 a

Different letters in each row indicate significant differences among the populations (paired bootstrap test, $P \leq 0.05$) using TWO-SEX MS chart program.

* Standard errors of means (SEM) were estimated using bootstrapping (100,000 re-samplings).

N = cohort size, d = days, rep. = reproductive, [a]adult pre-oviposition period, [b]total pre-oviposition period

Table 3 Demographic life table ~~traits~~ features of five house fly populations collected from dairies in Riyadh, Saudi Arabia

Parameters	Dirab ±SEM*	Al-Masanie ±SEM*	Al-Washlah ±SEM*	Al-Uraija ±SEM*	Al-Muzahmiya ±SEM*
intrinsic rate of increase (r) (d^{-1})	0.17 ± 0.01 a	0.13 ± 0.01 b	0.12 ± 0.02 b	0.18 ± 0.01 a	0.19 ± 0.01 a
finite rate of increase (λ) (d^{-1})	1.18 ± 0.01 a	1.13 ± 0.02 b	1.13 ± 0.02 b	1.19 ± 0.02 a	1.21 ± 0.01 a
net reproductive rate (R_0) (offspring/individual)	74.50 ± 21.45 ab	39.52 ± 13.88 b	33.04 ± 12.15 b	64.35 ± 17.40 ab	89.32 ± 20.56 a
mean generation time (T) (d)	26.05 ± 0.48 b	29.43 ± 0.89 a	29.80 ± 1.48 a	23.54 ± 0.47 c	24.08 ± 0.49 c
doubling time (DT) (d)	4.19 ± 0.34 ab	5.55 ± 1.07 ab	5.91 ± 1.06 a	3.92 ± 0.31 b	3.72 ± 0.24 b
gross reproduction rate (GRR) (offspring/individual)	305.78 ± 82.98 a	250.15 ± 79.20 a	240.46 ± 75.03 a	252.04 ± 64.02 a	344.18 ± 53.57 a

Different letters in each row indicate significant differences among the populations (paired bootstrap test, $P \leq 0.05$) using TWO-SEX MS chart program. *Standard errors of means (SEM) were estimated using bootstrapping (100,000 re-samplings).
d = days~~, *Standard errors of means were assessed by the bootstrap method with 100,000 replicates~~

Formatted: Justified

Formatted: Not Superscript/ Subscript

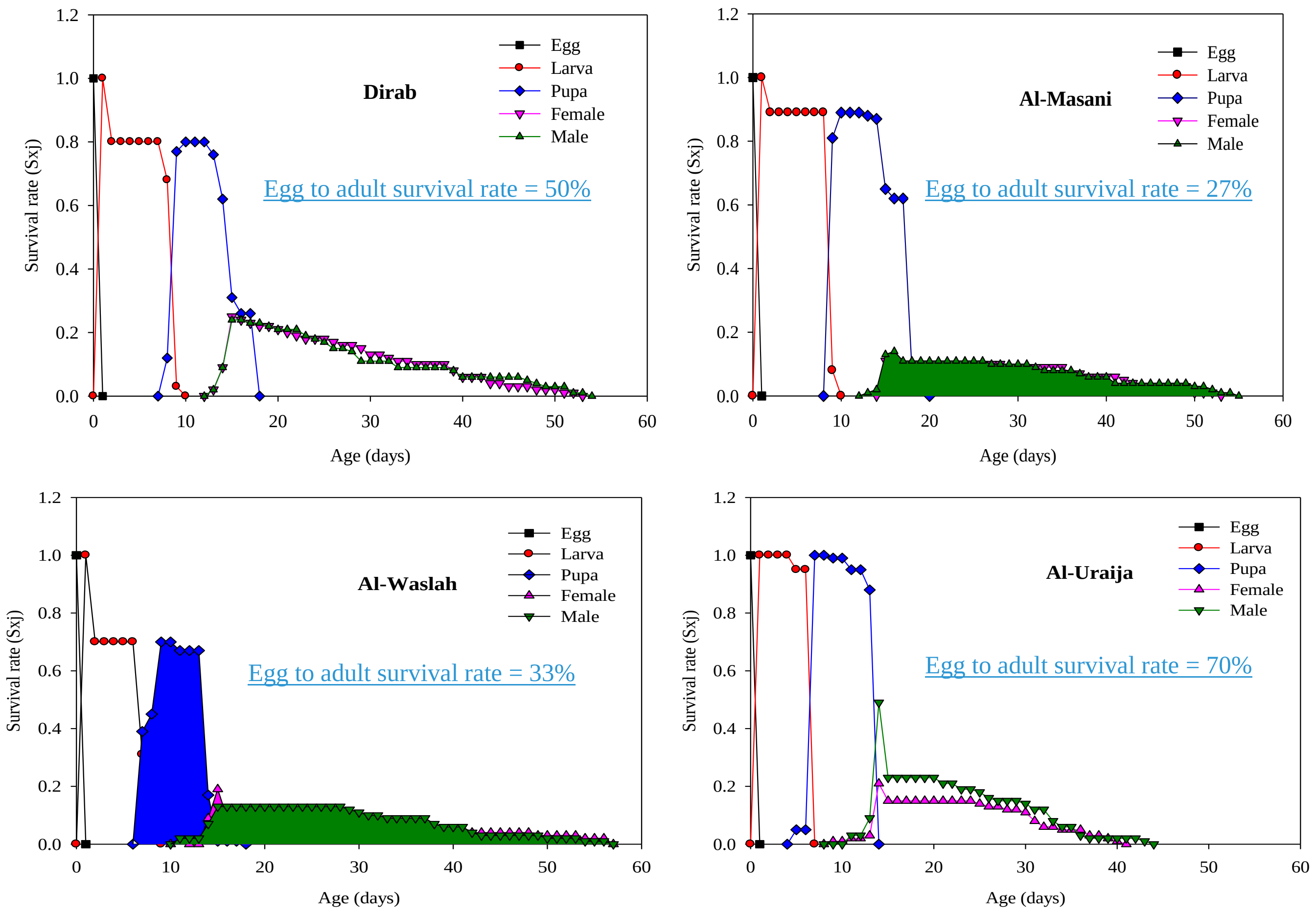
Dirab
Egg
Larva
Pupa
Female
Male
Egg to adult survival rate = 50%
Survival rate (Sxj)
Age (days)
Al-Masani
Egg to adult survival rate = 27%
Al-Waslah
Egg to adult survival rate = 33%
Al-Uraija
Egg to adult survival rate = 70%

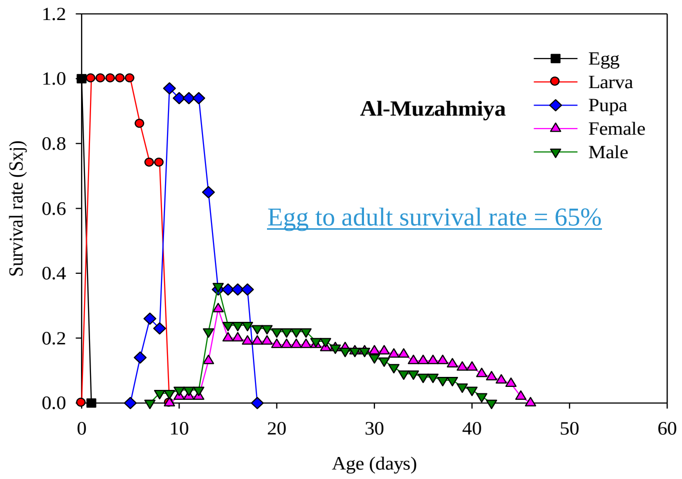

Fig. 1 Age-stage-specific survival rate (s_{xj}) for the Dirab, Al-Masanie, Al-Washlah, Al-Uraija, and Al-Muzahmiya populations of house fly.

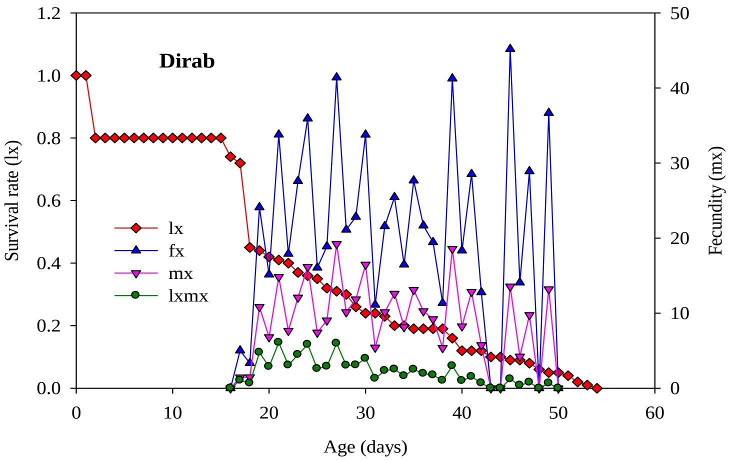
Dirab
Survival rate (lx)
Fecundity (mx)
Age (days)
lx
fx
mx
lxmx

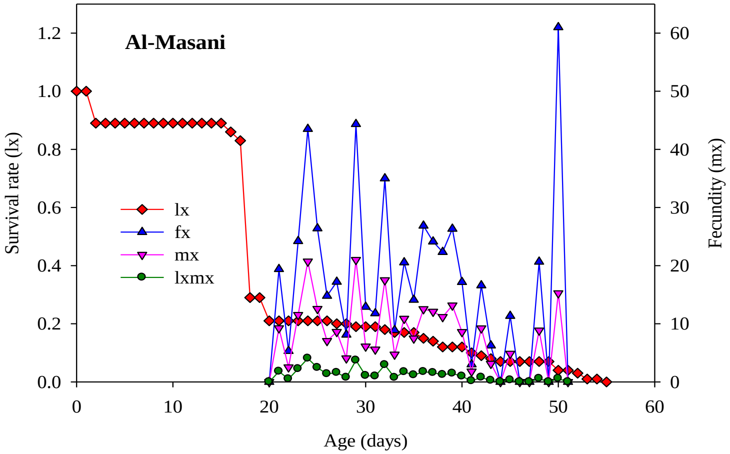
Al-Masani
Survival rate (lx)
Fecundity (mx)
Age (days)
lx
fx
mx
lxmx

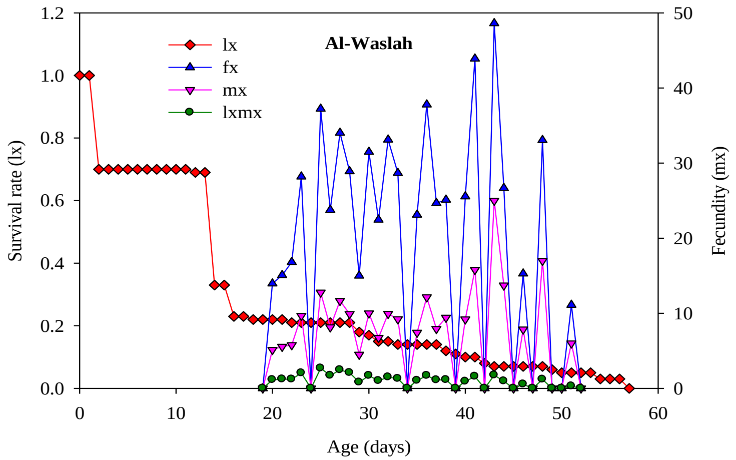
Al-Waslah
lx
fx
mx
lxmx
Survival rate (lx)
Fecundity (mx)
Age (days)
1.2
1.0
0.8
0.6
0.4
0.2
0.0
50
40
30
20
10
0
0
10
20
30
40
50
60

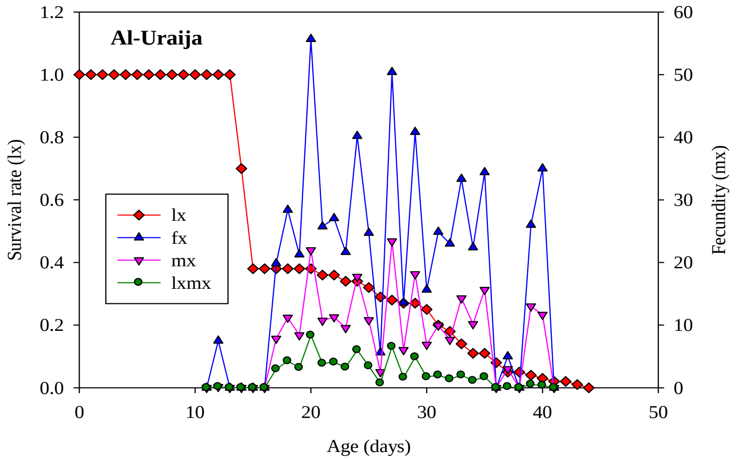
Al-Uraija
lx
fx
mx
lxmx
Survival rate (lx)
Fecundity (mx)
Age (days)
1.2
1.0
0.8
0.6
0.4
0.2
0.0
60
50
40
30
20
10
0
0
10
20
30
40
50

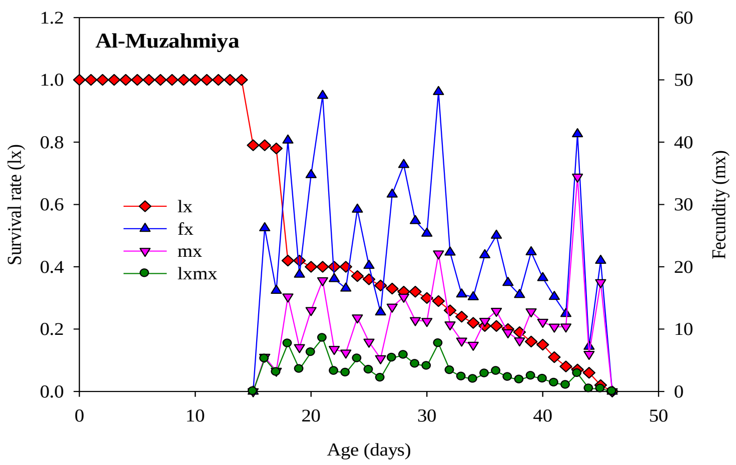

Fig. 2 Age-specific survival rate (l_x), age-specific female fecundity (f_x), age-specific fecundity of the total population (m_x), and age-specific maturity ($l_x m_x$) for the Dirab, Al-Masanie, Al-Washlah, Al-Uraija, and Al-Muzahmiya populations of house fly.

Formatted: Space After: 0 pt

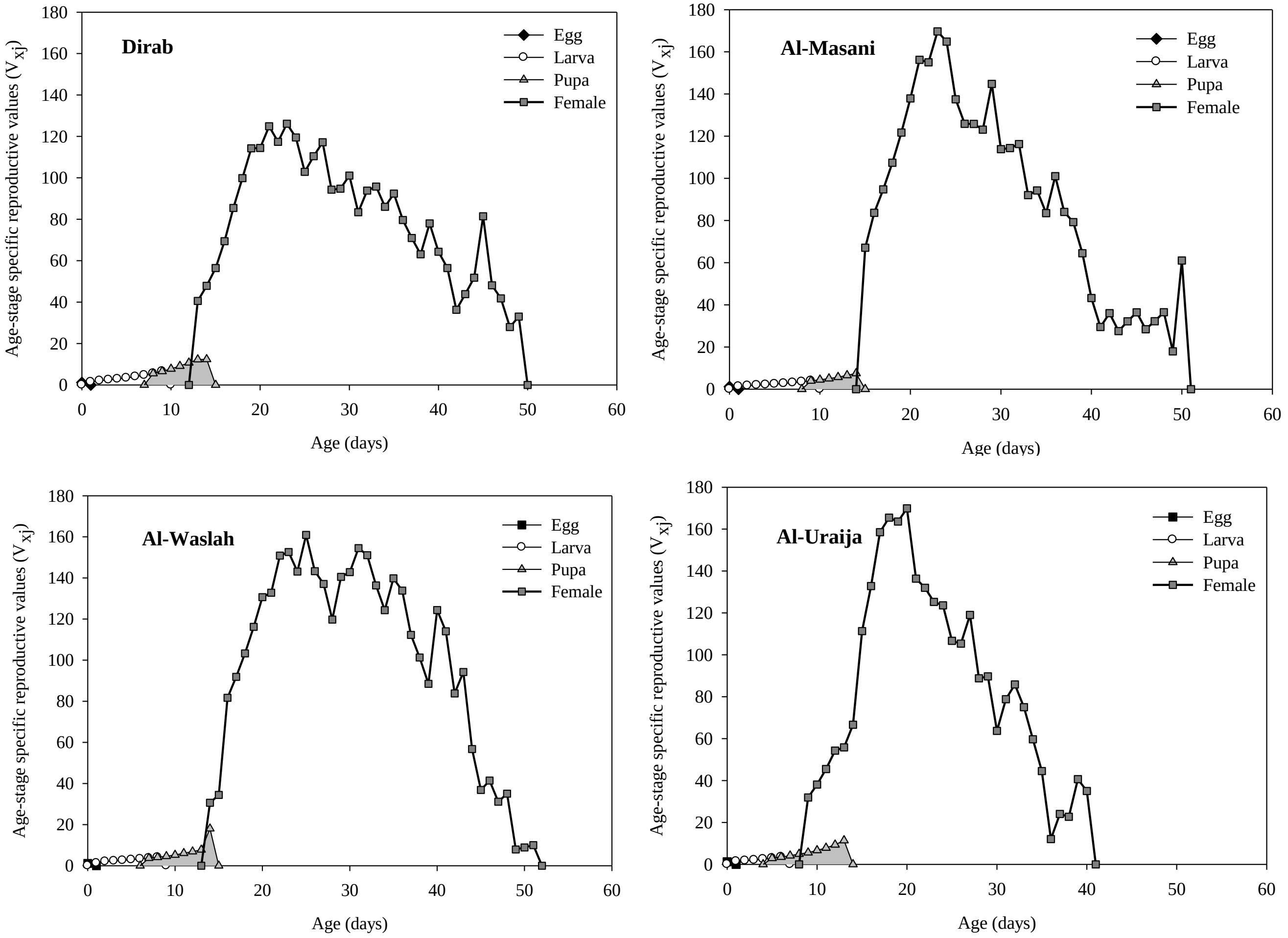
Dirab
Al-Masani
Al-Waslah
Al-Uraija
Egg
Larva
Pupa
Female
Age-stage specific reproductive values (V_{xj})
Age (days)

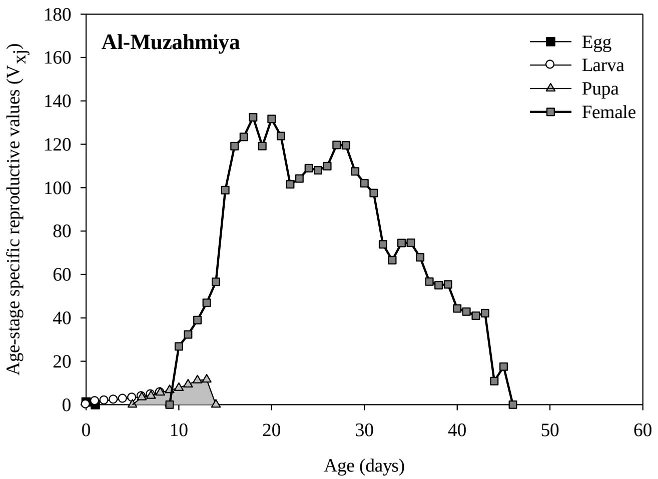

Fig. 3 Age-stage-specific reproductive values (v_{xj}) for the Dirab, Al-Masanie, Al-Washlah, Al-Uraija, and Al-Muzahmiya populations of house fly.

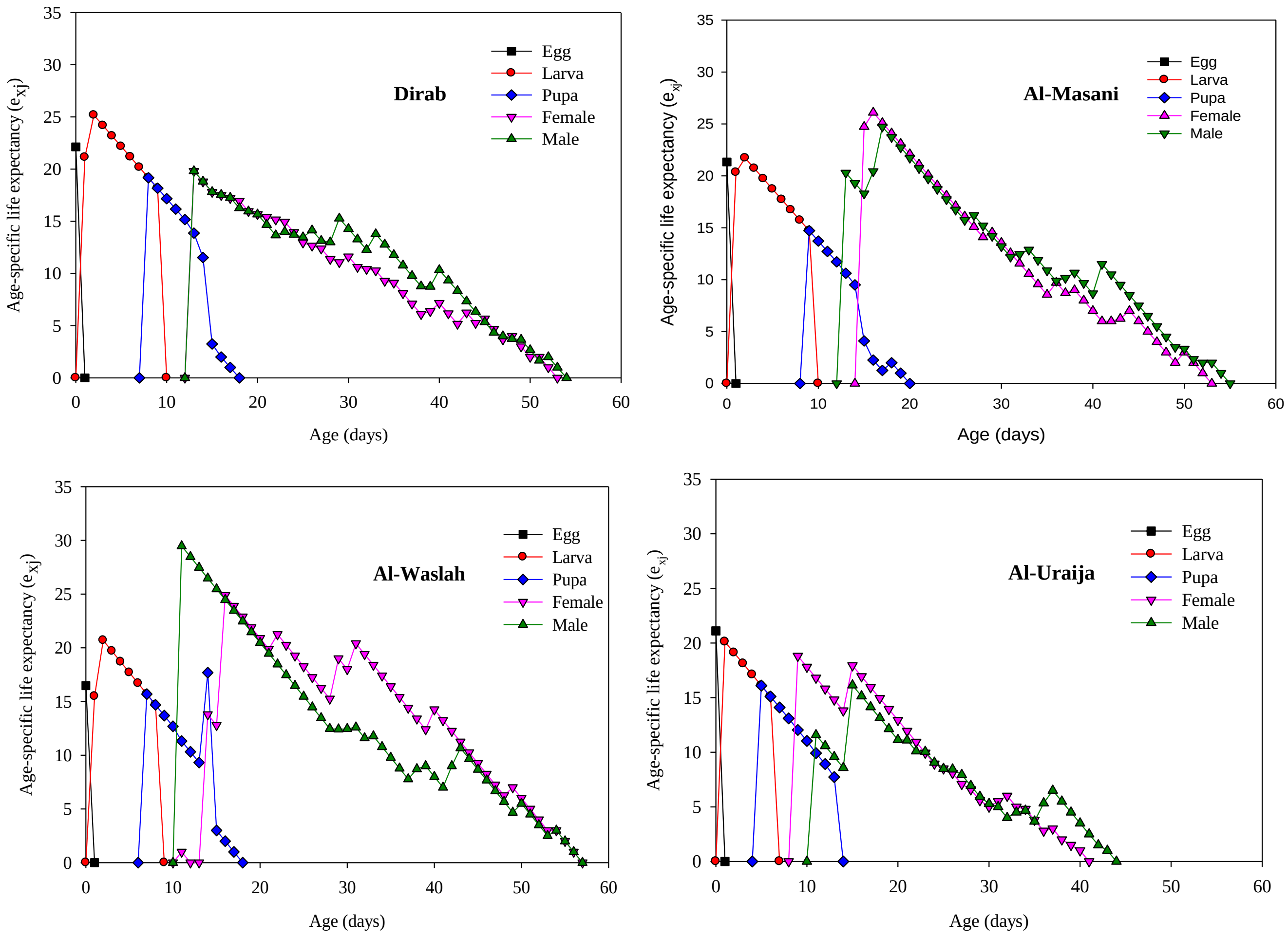
Dirab
Al-Masani
Al-Waslah
Al-Uraija
Egg
Larva
Pupa
Female
Male
Age-specific life expectancy (e_{xj})
Age (days)

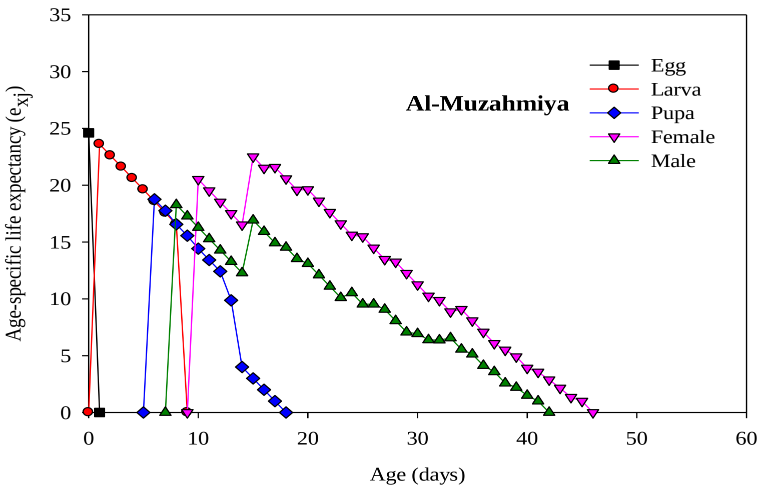

Fig. 4 Age-specific life expectancy (e_{xj}) for the Dirab, Al-Masanie, Al-Washlah, Al-Uraija, and Al-Muzahmiya populations of house fly.

Supplementary files

S1 Life table of *Musca domestica* population collected from Dirab, Saudi Arabia

S2 Life table of *Musca domestica* population collected from Al-Masanie, Saudi Arabia

S3 Life table of *Musca domestica* population collected from Al-Washlah, Saudi Arabia

S4 Life table of *Musca domestica* population collected from Al-Uraija, Saudi Arabia

S5 Life table of Musca domestica population collected from Al-Muzahmiya, Saudi Arabia

Formatted: Font: Not Bold
Formatted: Font: Not Bold, Italic
Formatted: Font: Not Bold
Formatted: Font: Not Bold
Formatted: Font: Not Bold
Formatted: Font: Not Bold, Italic
Formatted: Font: Not Bold
Formatted: Font: Not Bold
Formatted: Font: Not Bold
Formatted: Font: Not Bold, Italic
Formatted: Font: Not Bold
Formatted: Font: Not Bold
Formatted: Font: Not Bold
Formatted: Font: Not Bold, Italic
Formatted: Font: Not Bold
Formatted: Font: Not Bold
