# Supplementary material for: Resistance to insect growth regulators and age-stage, two-sex life table in Musca domestica from different dairy facilities
Source: PLoS One. 2021 Apr 8;16(4):e0248693. doi: 10.1371/journal.pone.0248693 (PMC8031857; doi:10.1371/journal.pone.0248693)
Supplement: Attachment — Submitted filename: Review report PONE-D-20-31395.docx [file pone.0248693.s006.docx]

**Review report:**

**Manuscript Number:** PONE-D-20-31395

**Full Title:** Resistance to insect growth regulator insecticides and age-stage, two-sex life table

parameters in house fly, Musca domestica from different dairy facilities

Comments:

The topic of the article is interesting and bears implications in management of housefly (*Musca domestica*) on a worldwide scale. Although the present study considered the housefly from dairy facilities, still, the information is useful for the population regulation of housefly elsewhere where the insecticide resistance is a problem. The study design including the experiments and statistical analyses and the compilation appears to be suitable for addressing the issues of insecticide resistance of housefly. However, few minor corrections are required with reference to the language and the title of the article.

Suggestions:

1] Please consider a concise title of this article.

2]The hypothesis of your study should be mentioned clearly at the end of the introduction section.

3]Please use an alternative to life table traits…you can consider life table features.

4]Please check for the typographical errors if any.

5] Please consider the following articles for inclusion in discussion section:

Kočišová A, Petrovský M, Toporčák J, Novák P. 2004. The potential of some insect growth regulators in housefly (*Musca domestica*) control. Biologia, 59/5: 661—668.

(Please consider this article as important to compare and highlight the impact of the insect growth regulators in housefly regulation. Please consider inclusion of the content of this paper in the introduction section.)

Malik A, Singh N, Satya S. 2007. House fly (*Musca domestica*): A review of control strategies for a challenging pest. Journal of Environmental Science and Health, Part B, 42:4, 453-469. [Please consider this article as important to highlight the various control measures available….both in the introduction and discussion sections]

Farooq M, Freed S. 2016. Infectivity of housefly, *Musca domestica* (Diptera: Muscidae) to different entomopathogenic fungi. Brazilian Journal of Microbiology, 47: 807 -816.

[ Please consider this article for highlighting the fungal agents in biocontrol of housefly]

Abbas N, & Khan A, Shad, S. 2014. Resistance of the house fly *Musca domestica* (Diptera: Muscidae) to lambda-cyhalothrin: Mode of inheritance, realized heritability, and cross-resistance to other insecticides. Ecotoxicology (London, England). 23. 10.1007/s10646-014-1217-7. [Please consider this article for the elaboration of resistance to pesticides by the housefly]

6] Please improve the quality of the figures, particularly, Figure 3.
