# Supplementary material for: Resistance to insect growth regulators and age-stage, two-sex life table in Musca domestica from different dairy facilities
Source: PLoS One. 2021 Apr 8;16(4):e0248693. doi: 10.1371/journal.pone.0248693 (PMC8031857; doi:10.1371/journal.pone.0248693)
Supplement: Attachment — Submitted filename: PONE-D-20-31395-general comments.docx [file pone.0248693.s008.docx]

29 January 2021

Plos One-my comments

**General comments:**

Although the authors have made several corrections based on the reviewers’ comments, there are still several concerns that I have mentioned both here and in the attached file. However, these amendments do not alleviate my fundamental concerns about the experimental design, validation, interpretation, and presentation of results.

This article deals with the investigation of resistance in five populations of houseflies to five IGR insecticides. The authors then try to explain the reasons behind this resistance to IGRs through life table studies. There are several main concerns that authors need to answer:

How can authors prove that houseflies collected from each area are a separate population? As the authors have presented in the methods, the coordinates of the sampling sites including geographical coordinates and altitude, this difference of locations cannot prove a significant effect on the fact that each is a separate population. With these coordinates, I cannot imagine a distinctive feature for each geographical area as a separate population. Also, houseflies can move easily between different areas in many ways.

The authors obtained the median lethal concentration (LC50) and resistance ratio (RR) for each IGR for each region and listed them in the table, but did not compare the correlation between LC50 values and RR of five IGRs in one region relative to the other regions. Or they have not examined the resistance of five regions to one IGR with another IGRs. In this manuscript, the authors provide only a simple descriptive report of IGR toxicity among areas. Therefore, it is not clear how the relationship of houseflies to IGRs in the regions are, and that based on this experiment it has not been answered whether each region can be considered a separate population or not. I became curious and calculated these correlational relationships, and as I expected, the populations' reaction to the IGRs was significantly correlated, and this result proves that they might be the same population.

In the following, the authors have only examined the life table of houseflies in each area. But they only reported the results of this study and no scientific interpretation has been provided behind it. It seems that the authors tried to interpret the reasons for the differences among the regions to the IGRs with the help of life table parameters, but did not achieve this goal. Although they have given a detailed report of the life table parameters in the results, the big issue with the discussion is they didn't really provide logical interpretation and validation of the results. Here the authors must examine the correlations between the main parameters of the life table with the LC50 and the RR, and provide a scientific, reasoned, and logical interpretation. The authors tried to state that the parameters of the life table have significant differences in different regions. It is usual to expect this because these differences are not so far from expectation. But that was not the purpose of the study at all.

**Specific comments:**

- line 24 and others: oviposition period

- line 46: give the full name of CHSI when first mentioned.

-line 60 and others: replace “L.” with “Linnaeus”. Keep uniformity.

Line 64: Replace F. with Fabricius.

Line 101 and others: replace mL with ml.

Line 131: newly laid eggs, do you mean £24 h??

Line 143 and others: use the word “survivorship” for lx. Use the word “survival rate” for Sxj.

Line 143-161: there are many errors in the formula of life table parameters that I have corrected in the attached file.

Line 377, 403, 407, 410, 413, 449, 453, 455, 476, 479, 513, and 544: Journal names should be written in the abbreviation that I have written the correct acronym for the journal in the attached file.
